# HIF-P4H-2 inhibition enhances intestinal fructose metabolism and induces thermogenesis protecting against NAFLD

**DOI:** 10.1007/s00109-020-01903-0

**Published:** 2020-04-15

**Authors:** Anna Laitakari, Joona Tapio, Kari A. Mäkelä, Karl-Heinz Herzig, Franziska Dengler, Helena Gylling, Gail Walkinshaw, Johanna Myllyharju, Elitsa Y. Dimova, Raisa Serpi, Peppi Koivunen

**Affiliations:** 1 Biocenter Oulu, Faculty of Biochemistry and Molecular Medicine, Oulu Center for Cell-Matrix Research, University of Oulu, Aapistie 7C, FIN-90014 Oulu, Finland; 2 Research Unit of Biomedicine, Biocenter Oulu, Medical Research Center and University Hospital, Oulu, Finland; 3 Department of Gastroenterology and Metabolism, Poznan University of Medical Sciences, Poznan, Poland; 4 University of Leipzig, Leipzig, Germany; 5 Internal Medicine, University of Helsinki and Helsinki University Hospital, 00029 HUS Helsinki, Finland; 6 FibroGen, Inc., San Francisco, CA USA

**Keywords:** Fructose, HIF, Hypoxia response, Metabolism, NAFLD

## Abstract

**Abstract:**

Non-alcoholic fatty liver disease (NAFLD) parallels the global obesity epidemic with unmet therapeutic needs. We investigated whether inhibition of hypoxia-inducible factor prolyl 4-hydroxylase-2 (HIF-P4H-2), a key cellular oxygen sensor whose inhibition stabilizes HIF, would protect from NAFLD by subjecting HIF-P4H-2-deficient (*Hif-p4h-2*^*gt/gt*^) mice to a high-fat, high-fructose (HFHF) or high-fat, methionine-choline-deficient (HF-MCD) diet. On both diets, the *Hif-p4h-2*^*gt/gt*^ mice gained less weight and had less white adipose tissue (WAT) and its inflammation, lower serum cholesterol levels, and lighter livers with less steatosis and lower serum ALT levels than the wild type (WT). The intake of fructose in majority of the *Hif-p4h-2*^*gt/gt*^ tissues, including the liver, was 15–35% less than in the WT. We found upregulation of the key fructose transporter and metabolizing enzyme mRNAs, *Slc2a2*, *Khka*, and *Khkc*, and higher ketohexokinase activity in the *Hif-p4h-2*^*gt/gt*^ small intestine relative to the WT, suggesting enhanced metabolism of fructose in the former. On the HF-MCD diet, the *Hif-p4h-2*^*gt/gt*^ mice showed more browning of the WAT and increased thermogenesis. A pharmacological pan-HIF-P4H inhibitor protected WT mice on both diets against obesity, metabolic dysfunction, and liver damage. These data suggest that HIF-P4H-2 inhibition could be studied as a novel, comprehensive treatment strategy for NAFLD.

**Key messages:**

• HIF-P4H-2 inhibition enhances intestinal fructose metabolism protecting the liver.

• HIF-P4H-2 inhibition downregulates hepatic lipogenesis.

• Induced browning of WAT and increased thermogenesis can also mediate protection.

• HIF-P4H-2 inhibition offers a novel, comprehensive treatment strategy for NAFLD.

**Electronic supplementary material:**

The online version of this article (10.1007/s00109-020-01903-0) contains supplementary material, which is available to authorized users.

## Introduction

The prevalence of non-alcoholic fatty liver disease (NAFLD) is constantly increasing, currently affecting a quarter of people worldwide [1]. It is considered the hepatic manifestation of metabolic syndrome, strongly linked to obesity and insulin resistance (IR), which further predisposes for diabetes and cardiovascular diseases [2]. NAFLD is characterized by hepatic triglyceride accumulation and if untreated, can lead to steatohepatitis (NASH), cirrhosis, and hepatocellular carcinoma (HCC) [1]. Western diet, rich in saturated fat and carbohydrates, especially fructose, is considered to be one of the major causes of the NAFLD epidemic [3]. No effective cure is currently available [4].

A decrease in oxygen availability activates a survival mechanism called the hypoxia response [5–7]. Hypoxia-inducible factor (HIF) functions as the major regulator of oxygen homeostasis, and HIF prolyl 4-hydroxylases (HIF-P4Hs/PHDs/EglNs), especially HIF-P4H-2, are oxygen sensors that target HIFα for degradation under normoxia [5, 8]. Under hypoxia, the oxygen-dependent hydroxylation is compromised [9], allowing HIF to form a transcriptionally active αβ-dimer and upregulate > 300 genes. These genes increase oxygen availability by inducing erythropoiesis and angiogenesis, and also reduce its demand via regulation of energy metabolism by reducing oxidative phosphorylation and inducing non-oxygen-demanding glycolysis [5–7].

The first-in-class small-molecule HIF-P4H inhibitor that activates the hypoxia response has been approved for treatment of renal anemia and several others are in clinical trials [10, 11]. Recent data suggest that besides anemia, HIF-P4H inhibition and hypoxia are powerful tools for promoting metabolic health [10, 12–17]. We have previously shown that genetic HIF-P4H-2 deficiency protects the *Hif-p4h-2*^*gt/gt*^ mice against metabolic disorder–related hepatic steatosis and chemically induced HCC [14, 18], and against alcoholic liver disease (ALD) by downregulating hepatic lipogenesis and improving the elimination of harmful ethanol metabolites and reactive oxygen species [19]. Treatment of wild-type (WT) mice with a pan-HIF-P4H inhibitor FG-4497 phenocopied the protection against ALD [19]. However, the role of HIF-P4H-2 in NAFLD has not been studied systemically before. We therefore subjected the *Hif-p4h-2*^*gt/gt*^ mice, and FG-4497-treated WT mice, to two diet-induced rodent NAFLD models: a high-fat, high-fructose (HFHF) diet [20] and a methionine-choline-deficient high-fat diet (HF-MCD) [21] that mimic the human disease. The HF-MCD can additionally lead to NASH [22]. Our data show significantly less steatosis and liver damage in the *Hif-p4h-2*^*gt/gt*^ mice compared with the WT. Treatment with FG-4497 phenocopied most of these effects.

## Materials and methods

### Animal experiments

Animal experiments were performed according to protocols approved by the National Animal Experiment Board of Finland (ESAVI-6154, ESAVI-8179). *Hif-p4h-2*^*gt*/*gt*^ mice were generated as previously described [23]. Five-month-old *Hif-p4h-2*^*gt/gt*^ and WT males were fed a 30% (w/v) fructose solution for drinking water combined with a high-fat, modified Surwit diet with added cholesterol (HFHF diet, D09061703, 58% kcal fat) for 8 weeks. Six-month-old and 2-month-old *Hif-p4h-2*^*gt/gt*^ and WT females were fed a high-fat, choline-deficient diet with 0.1% methionine (HF-MCD diet, A06071309, 45% kcal fat) for 7 weeks, and the 2-month-old mice were studied with an automated home cage phenotyping system (PhenoMaster, TSE Systems) for the last week. For the pharmacological studies, 8-month-old WT females (*Hif-p4h-2*^*gt/gt*^ littermates) were fed the HFHF diet for 6 weeks and 4-month-old WT females (C57BL/6JRccHsd, Envigo) the HF-MCD diet for 3 weeks and given thrice a week orally 60 mg/kg FG-4497 (FibroGen, Inc., USA) or vehicle.

Further methods are described in the supplementary material.

## Results

### HIF-P4H-2-deficient mice were protected from fructose diet–induced weight gain, but the diet did not induce NAFLD

*Hif-p4h-2*^*gt/gt*^ mice and their WT littermates were fed a standard rodent diet with a 30% fructose solution for 16 weeks. Although the daily intake of the fructose solution was similar between the genotypes (Fig. S1a), the *Hif-p4h-2*^*gt/gt*^ mice had a ~ 20% lower body weight than the WT at sacrifice, had gained less weight during the diet, and had > 50% less gonadal white adipose tissue (WAT) (Fig. S1b–d). The *Hif-p4h-2*^*gt/gt*^ mice also showed a slight trend towards better glucose tolerance compared with the WT (Fig. S1e). The *Hif-p4h-2*^*gt/gt*^ livers were 21% lighter than the WT livers (Fig. S1f), suggesting more fructose-induced hepatic steatosis in the WT, since no baseline difference exits between the genotypes [14]. However, the diet only induced visible steatosis and increased the serum alanine aminotransferase (ALT) levels in a few WT mice, and no liver inflammation in either genotype (Fig. S1g–i), thus not being potent enough to induce NAFLD. Hence, we next combined the 30% fructose solution with a high-fat diet (HFHF) to better mimic Western diet.

### HIF-P4H-2-deficient mice were protected from obesity and retained a healthier serum lipid profile than the WT on a HFHF diet

**Fig. 1:**
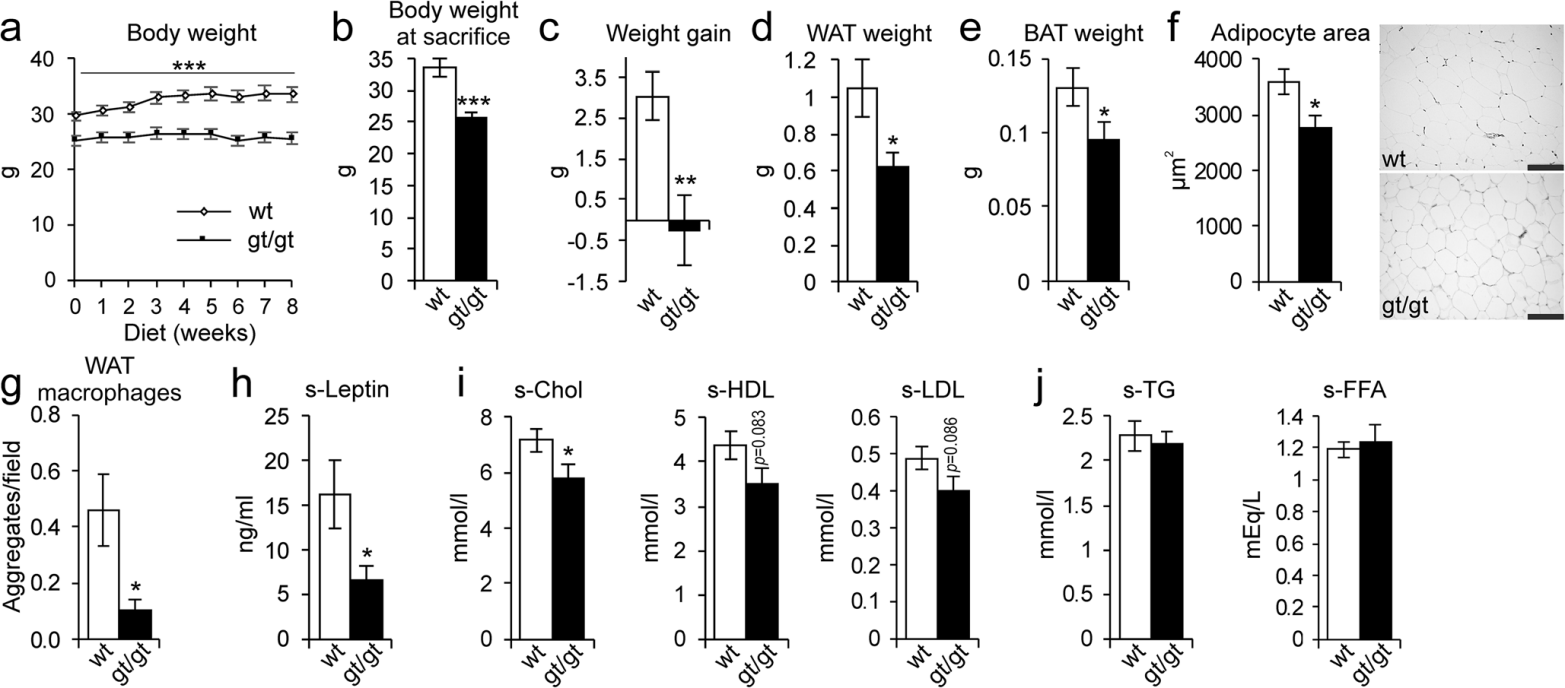
HIF-P4H-2-deficient mice are protected against high-fat, high-fructose (HFHF) diet–induced obesity, adipose tissue inflammation, and high cholesterol levels. Wild-type (wt) and Hif-p4h-2^*gt/gt*^ (gt/gt) males were studied while on an 8-week HFHF diet (*n* = 8–10/group). **a** Body weight development during the diet. **b** Body weight at sacrifice. **c** Weight gain at the end of the diet relative to weights on the day before the diet started. **d** Weight of gonadal WAT. **e** Weight of BAT. **f** Cross-sectional area of WAT adipocytes. Scale bar = 100 μm. **g** Number of macrophage aggregates in WAT. **h** Serum leptin levels. **i** Serum total cholesterol, HDL cholesterol, and LDL cholesterol levels. **j** Serum TG and FFA levels (*n* = 6–10/group for FFA). Data are means ± SEM. *p < 0.05, **p < 0.01, ***p < 0.001. BAT, brown adipose tissue; FFA, free fatty acids; s, serum; TG, triglycerides; WAT, white adipose tissue

The *Hif-p4h-2*^*gt/gt*^ and WT mice were fed the HFHF diet for 8 weeks, during which the former did not gain any weight, whereas the latter’s body weight increased by ~ 10%, resulting in an almost 30% higher body weight at sacrifice (Fig. 1a–c). Consistently with this, the *Hif-p4h-2*^*gt/gt*^ mice had 40% less WAT and also less brown adipose tissue (BAT) than the WT (Fig. 1d, e), as well as smaller adipocytes (Fig. 1f). Additionally, the *Hif-p4h-2*^*gt/gt*^ mice had less inflammatory macrophage aggregates in their WAT than the WT (Fig. 1g), and furthermore, their serum leptin levels were lower (Fig. 1h). The HFHF diet induced elevation of the serum total cholesterol, HDL, and LDL levels in the WT, which the *Hif-p4h-2*^*gt/gt*^ mice were protected against (Fig. 1i). There were no differences in serum triglyceride and free fatty acid (FFA) levels between the genotypes (Fig. 1j).

### *Hif-p4h-2*^*gt/gt*^ mice were protected against HFHF diet–induced steatosis and liver damage

**Fig. 2:**
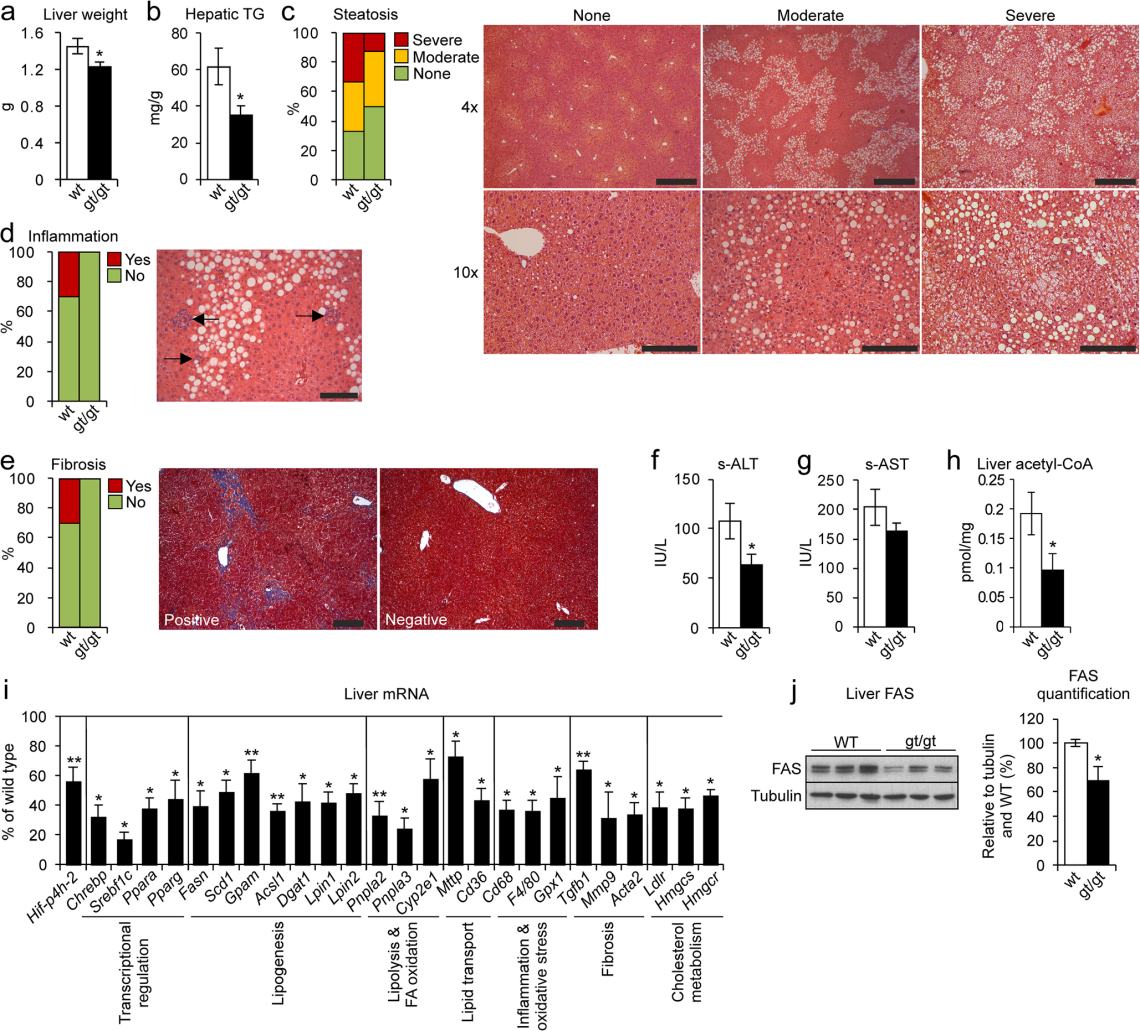
HIF-P4H-2 deficiency protects mice from high-fat, high-fructose (HFHF) diet–induced fatty liver disease. Wild-type (wt) and Hif-p4h-2^gt/gt^ (gt/gt) males were studied while on an 8-week HFHF diet (*n* = 8–10/group). **a** Liver weight. **b** Hepatic triglyceride content. **c** Scoring of steatosis and H&E-stained liver sections. Steatosis grading: “None” corresponds to scores 0–2, “Moderate” to 3, and “Severe” to 4. Images are representative of scoring for wt. Scale bar = 500 μm for × 4 and 200 μm for × 10. **d** Scoring of inflammation from H&E-stained liver sections. “No” corresponds to score 0 and “Yes” to 1–2. Arrows indicate neutrophil clusters in wt. Scale bar = 100 μm. **e** Scoring of fibrosis from Masson’s trichrome–stained liver sections. “No” corresponds to score 0 and “Yes” to 1–2. Images are representative of scoring for wt. Scale bar = 100 μm. **f** Serum ALT levels. **g** Serum AST levels. **h** Liver acetyl-CoA levels. **i** qPCR analysis of liver mRNA levels of gt/gt mice relative to wt, studied relative to TATA-box-binding protein mRNA. **j** Western blotting and densitometric quantification of hepatic FAS levels (*n* = 4/group.). Tubulin was used as a loading control. **a**, **b**, **f**–**j** Data are means ± SEM. *p ≤ 0.05, **p < 0.01. ACSL1, acyl-CoA synthetase long-chain family member 1; ACTA2, actin alpha 2, smooth muscle; ALT, alanine aminotransferase; AST, aspartate aminotransferase; CHREBP, carbohydrate-responsive element-binding protein; CYP2E1, cytochrome P450 family-2 subfamily e member 1; DGAT1, diacylglycerol o-acyltransferase 1; FAS/FASN, fatty acid synthase; FA, fatty acid; GPAM, mitochondrial glycerol-3-phosphate acyltransferase; GPX1, glutathione peroxidase 1; HMGCS/HMGCR, hydroxymethylglutaryl-CoA synthase/reductase; LDLR, low-density lipoprotein receptor; LPIN, lipin; MMP9, matrix metallopeptidase 9; MTTP, microsomal triglyceride transfer protein; PNPLA, patatin-like phospholipase domain containing; PPAR a/g, peroxisome proliferator–activated receptor alpha/gamma; s, serum; SCD1, stearoyl-CoA desaturase 1; SREBF1c, sterol regulatory element-binding protein 1c; TG, triglycerides; TGFb1, transforming growth factor beta 1

After the 8-week HFHF diet, the WT liver weights were significantly ~ 16% higher than the *Hif-p4h-2*^*gt/gt*^ (Fig. 2a), and consistently, the hepatic triglyceride content of the *Hif-p4h-2*^*gt/gt*^ livers was > 40% less than that of the WT (Fig. 2b). Histological evaluation demonstrated that 50% of the *Hif-p4h-2*^*gt/gt*^ livers had no steatosis, compared with 33% of the WT, while severe steatosis was observed in > 30% of the WT, but only 10% of the *Hif-p4h-2*^*gt/gt*^ livers (Fig. 2c). The HFHF diet also induced mild inflammation and fibrosis in ~ 30% of the WT livers but not the *Hif-p4h-2*^*gt/gt*^ (Fig. 2d, e). The serum ALT levels in the *Hif-p4h-2*^*gt/gt*^ mice were slightly above the physiological limit (55 IU/L) whereas the WTs were at a pathological level of 107 IU/L (Fig. 2f). Similar trend was observed in the serum aspartate aminotransferase (AST) levels (Fig. 2g). Additionally, lower levels of hepatic acetyl-CoA, lipid, and cholesterol biosynthesis precursor were observed in the *Hif-p4h-2*^*gt/gt*^ livers than in the WT (Fig. 2h). In agreement, many hepatic lipid metabolism mRNAs were downregulated in the *Hif-p4h-2*^*gt/gt*^ livers compared with those in WT. These included the key regulators of glucose metabolism and lipid synthesis, the glucose-activated *Chrebp* and the insulin-activated *Srebf1c*, and their target genes *Fasn*, *Scd1*, and *Gpam*; the master regulators of hepatic lipid metabolism *Ppara* and *Pparg* along with their target genes *Acsl1*, *Dgat1*, *Pnpla2*, *Pnpla3*, *Mttp*, and *Cd36*; and the lipogenesis, fatty acid (FA) oxidation, and cholesterol metabolism mRNAs *Lpin1*, *Lpin2, Cyp2e1*, *Ldlr*, *Hmgcr*, and *Hmgcs* (Fig. 2i). Furthermore, the inflammation and fibrosis markers *Cd68*, *F4/80*, *Tgfb1*, *Mmp9*, and *Acta2* (*α-sma*) were downregulated, corresponding to the histological findings, as was *Gpx1*, whose downregulation in mouse liver improves glucose metabolism and reduces steatohepatitis (Fig. 2i) [24, 25]. In agreement, a 30% decrease was observed in the protein levels of FAS in the *Hif-p4h-2*^*gt/gt*^ livers (Fig. 2j). The hepatic *Hif-p4h-2* mRNA levels were down to a similar extent (~ 40%) as reported earlier [14] (Fig. 2i), and the protein levels of HIF-P4H-2, studied with baseline *Hif-p4h-2*^*gt/gt*^ and WT primary hepatocytes, were correspondingly downregulated (Fig. S2). Altogether, these data indicate that the *Hif-p4h-2*^*gt/gt*^ mice were protected from HFHF diet–induced metabolic symptoms of NAFLD, hepatic fat accumulation, and liver damage.

### HIF-P4H-2-deficient mice had lower hepatic fructose levels and better glucose tolerance on the HFHF diet than the WT

**Fig. 3:**
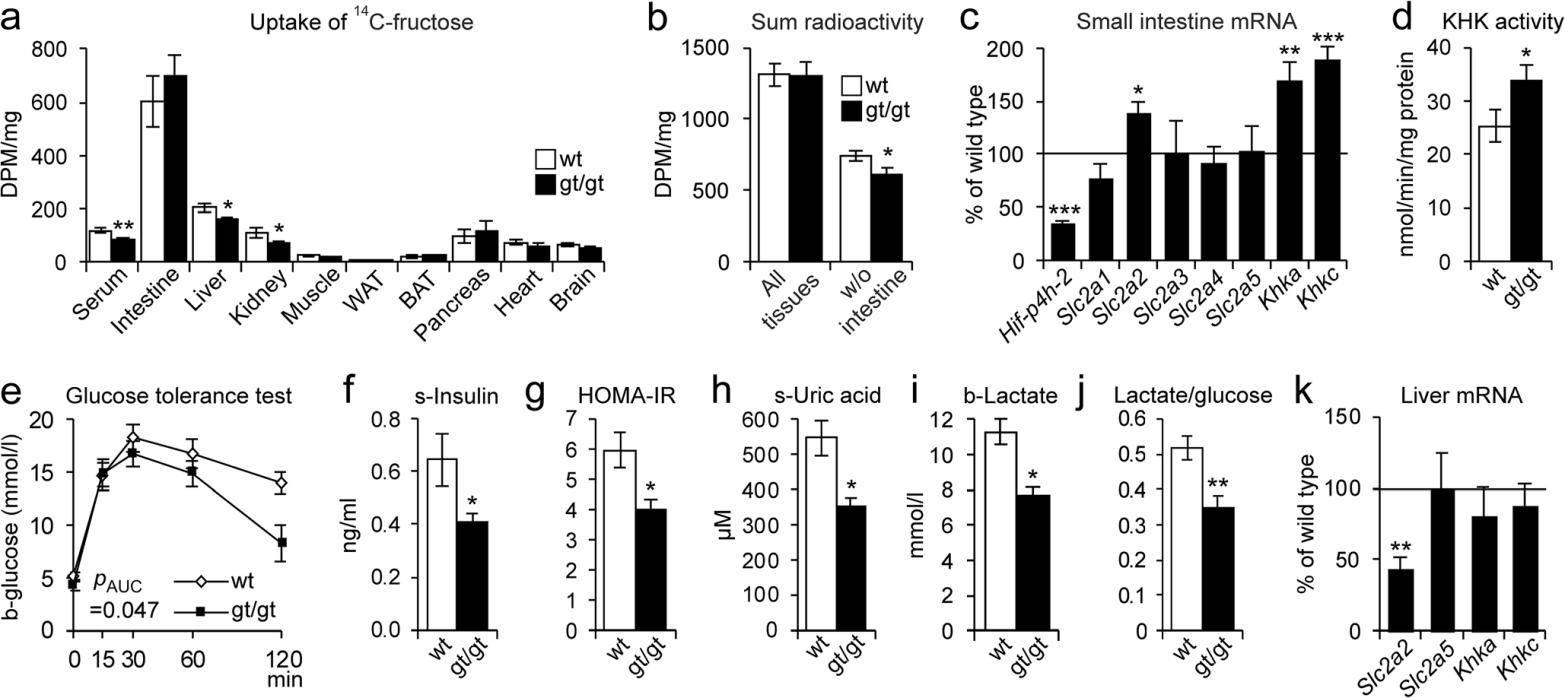
Changes in fructose metabolism associated with protection from high-fat, high-fructose (HFHF) diet–induced NAFLD in the HIF-P4H-2-deficient mice. Wild-type (wt) and Hif-p4h-2^gt/gt^ (gt/gt) males after **e**–**g** 6 or **c**, **h**–**k** 8 weeks on a HFHF diet (*n* = 8–10/group). **a**, **b**^14^C-fructose uptake test. After 2 weeks on the HFHF diet and a 12-h fast, wt and gt/gt females received ^14^C-fructose orally and were sacrificed after 15 min and tissue samples were measured for ^14^C radioactivity as disintegration/min/mg (DPM) (*n* = 12/group). **a** DPM per mg of tissue in indicated tissues. **b** Sum radioactivity detected in all tissues measured including and excluding the small intestine. **c** qPCR analysis of the mRNA levels of glucose and fructose transporters and metabolizing enzymes in the small intestine of the gt/gt mice relative to wt. Gene expression was studied relative to β-actin mRNA. **d** Ketohexokinase activity in the small intestine of females after 4 weeks on a HFHF diet (*n* = 6–8/group). **e** Oral glucose tolerance test (GTT). The value for 0 min was determined after a 12-h fast. **f** Serum insulin levels determined from the 0-min GTT samples. **g** HOMA-IR scores determined from the 0-min values. **h** Serum uric acid levels. **i** Blood lactate levels. **j** Blood lactate-to-glucose ratio. **k** qPCR analysis of liver mRNA levels of glucose and fructose transporters and metabolizing enzymes in gt/gt mice relative to wt, studied relative to TATA-box-binding protein mRNA. Data are means ± SEM. *p < 0.05, **p < 0.01, ***p < 0.001. The *p* value for **d** was calculated from log-transformed values. b, blood; BAT, brown adipose tissue; HOMA-IR, homeostatic model assessment-insulin resistance; KHK, ketohexokinase; s, serum; SLC2A 1-5, solute carrier family-2 member 1-5; WAT, white adipose tissue

We next studied the ^14^C-labeled fructose uptake by tissues 15 min after oral administration. Majority of radioactivity was found in the intestine in both genotypes (Fig. 3a) while no difference was seen in its secretion to feces or urine between the genotypes (Fig. S3a). Interestingly, the ^14^C levels measured in most tissues were 15–35% lower in the *Hif-p4h-2*^*gt/gt*^ mice than in the WT, the reduction in the liver, kidney, and serum reaching significance, unlike the induction of ~ 20% in the intestine, pancreas, and BAT (Fig. 3a). The sum of radioactivity in all tissues was similar in both genotypes (Fig. 3b), but the sum excluding the intestine was significantly lower in the *Hif-p4h-2*^*gt/gt*^ mice, suggesting that the lower amount of fructose found in the liver, kidney, and serum would correspond to increased uptake by the *Hif-p4h-2*^*gt/gt*^ intestine (Fig. 3a). The small intestine metabolizes most of dietary fructose ketohexokinase (KHK) dependently, and if its clearance capacity is exceeded by high fructose concentration, the excess fructose spills over into the liver and other organs, as reported previously [26]. Therefore, we studied the mRNA levels of KHK isoenzymes and fructose transporters and found upregulation of *Slc2a2* (GLUT2) mRNA levels in the *Hif-p4h-2*^*gt/gt*^ small intestine relative to the WT, the downregulation of *Hif-p4h-2* mRNA being ~ 65% in the intestine in the former (Fig. 3c). However, no difference was seen in the immunostaining of GLUT2 (Fig. S3b). Interestingly, the *Khka* and *Khkc* mRNA levels were significantly higher in the *Hif-p4h-2*^*gt/gt*^ small intestine than in the WT (Fig. 3c) as was the measured catalytic KHK activity (Fig. 3d). Altogether, these data suggest enhanced fructose metabolism in the *Hif-p4h-2*^*gt/gt*^ intestine, which would result in less fructose being transported to the liver and other tissues.

Furthermore, the HFHF-fed *Hif-p4h-2*^*gt/gt*^ mice maintained a better glucose tolerance than the WT and had lower serum insulin levels and HOMA-IR scores, indicating better insulin sensitivity (Fig. 3e–g). A similar reduction was seen in circulating levels of the harmful byproducts of fructose metabolism, uric acid, and lactate (Fig. 3h, i), supporting differences in its metabolism between genotypes. This, and the lower lactate/glucose ratio in the *Hif-p4h-2*^*gt/gt*^ mice (Fig. 3j), agrees with their previously characterized enhanced lactate clearance [27]. The hepatic mRNA level of the fructose-uptaking *Slc2a2* was significantly lower in the *Hif-p4h-2*^*gt/gt*^ than in the WT, supporting the reduced hepatic ^14^C-fructose uptake, while there was no difference between the genotypes in the levels of *Slc2a5* or *Khk*s (Fig. 3k). Of note, the expression level of *Slc2a2* mRNA was ~65-fold compared *Slc2a5* (data not shown), being in agreement with the role of GLUT2 as the main hepatic fructose transporter.

### HIF-P4H-2-deficient mice were protected from obesity and retained a healthier serum lipid profile when challenged with an HF-MCD diet

**Fig. 4:**
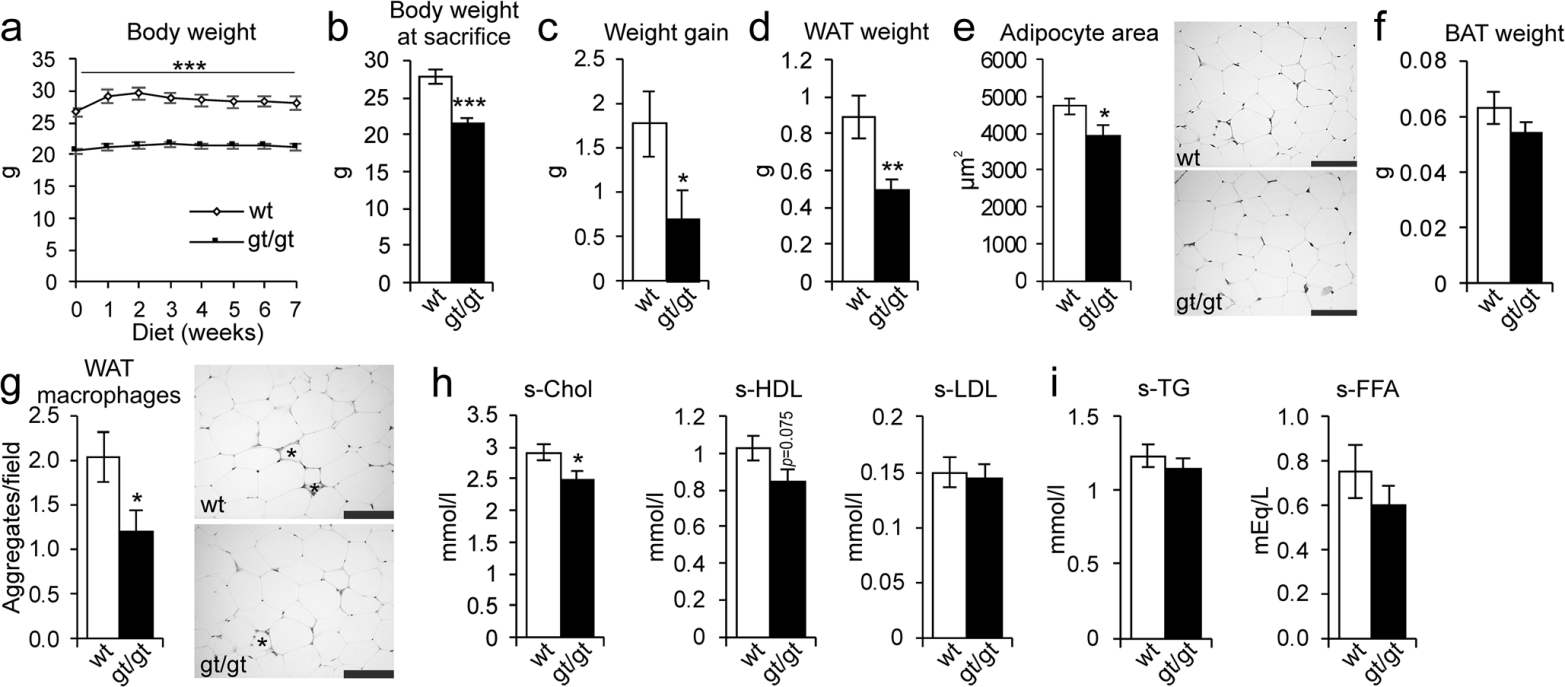
HIF-P4H-2-deficient mice are protected against HF-MCD diet–induced obesity, adipose tissue inflammation, and higher cholesterol levels. Wild-type (wt) and Hif-p4h-2^gt/gt^ (gt/gt) females were studied while on a 7-week high-fat, methionine-choline-deficient (HF-MCD) diet (*n* = 10–12/group). **a** Body weight development during the 7 weeks. **b** Body weight at sacrifice. **c** Weight gain at the end of the diet relative to weights on the day before the diet started. **d** Weight of gonadal WAT. **e** Cross-sectional area of WAT adipocytes. Scale bar = 100 μm. **f** Weight of BAT. **g** Number of macrophage aggregates in WAT. *Adipocytes surrounded by macrophage aggregates. Scale bar = 100 μm. **h** Serum total cholesterol, HDL cholesterol, and LDL cholesterol levels. **i** Serum TG and FFA levels (*n* = 6–9/group). Data are means ± SEM. *p < 0.05, **p < 0.01, ***p < 0.001. BAT, brown adipose tissue; FFA, free fatty acids; s, serum; TG, triglycerides; WAT, white adipose tissue

Next, the *Hif-p4h-2*^*gt/gt*^ and WT mice were fed the HF-MCD diet, inducing a more severe form of NAFLD than the HFHF diet. The weight gain of the *Hif-p4h-2*^*gt/gt*^ mice was significantly lower than that of the WT during the 7-week diet, and at sacrifice, they had a 22% lower body weight (Fig. 4a–c). In agreement, the *Hif-p4h-2*^*gt/gt*^ mice had 45% less WAT than the WT with significantly smaller adipocytes and a trend towards lower BAT weight (Fig. 4d–f). Also, less macrophage aggregates were detected in their WAT (Fig. 4g). Additionally, the *Hif-p4h-2*^*gt/*gt^ mice retained a healthier serum lipid profile than the WT, their total cholesterol level being significantly lower (Fig. 4h), the same applying to their glucose levels (Fig. S4). No differences in serum triglyceride and FFA levels were detected between the genotypes on this diet, either (Fig. 1i).

### *Hif-p4h-2*^*gt/gt*^ mice were protected against HF-MCD diet–induced steatosis and liver damage

**Fig. 5:**
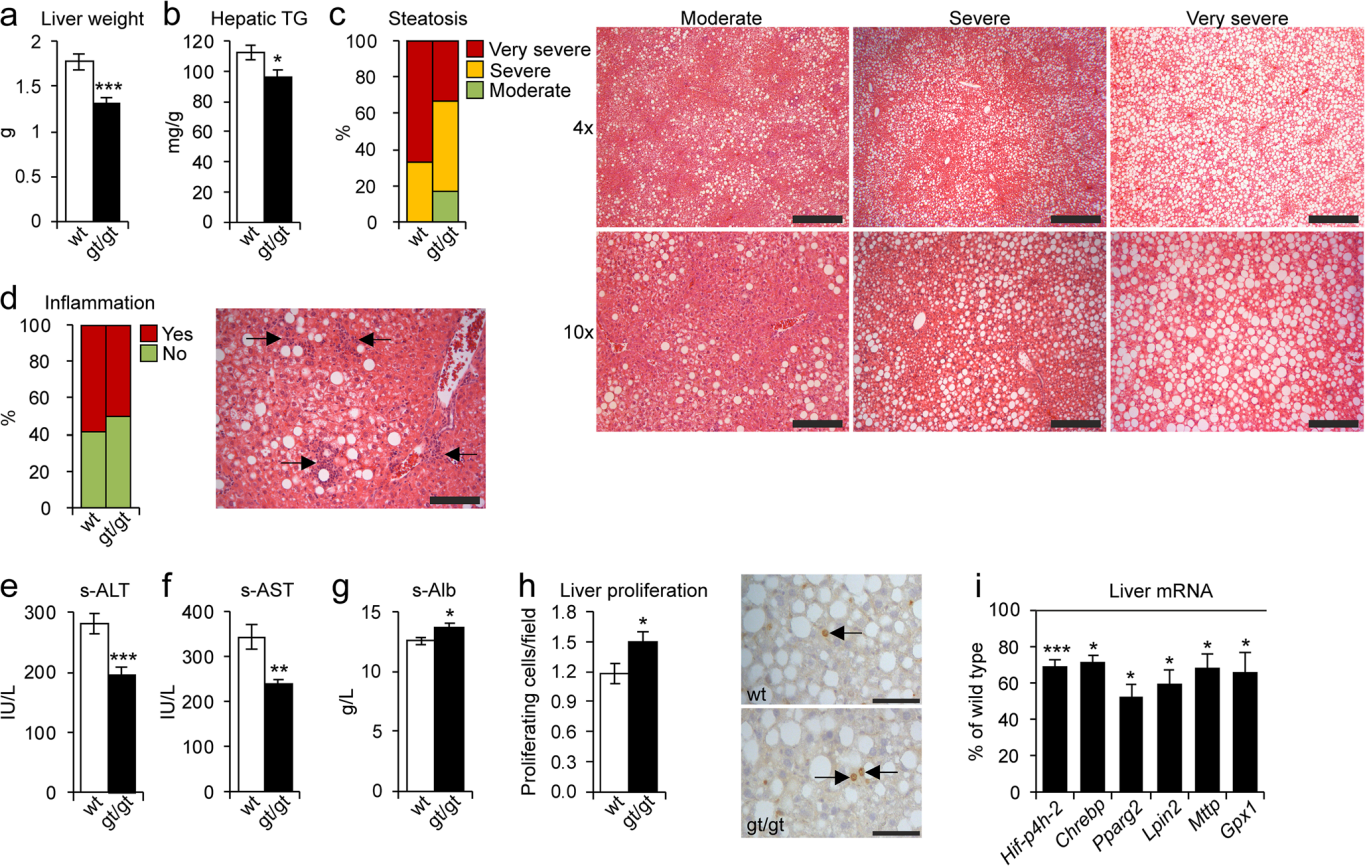
HIF-P4H-2 deficiency protects mice from HF-MCD diet–induced fatty liver disease. Wild-type (wt) and Hif-p4h-2^gt/gt^ (gt/gt) females were studied while on a 7-week high-fat, methionine-choline-deficient (HF-MCD) diet (*n* = 10–12/group). **a** Liver weight. **b** Hepatic triglyceride content. **c** Scoring of steatosis and H&E-stained liver sections. Steatosis grading: “Moderate” corresponds to score 2, “Severe” to 3, and “Very severe” to 4. “Very severe” and “severe” images are representative of scoring for wt, and “moderate” for gt/gt. Scale bar = 500 μm for × 4 and 200 μm for × 10. **d** Scoring of inflammation from H&E-stained liver sections. “No” corresponds to scores 0–1 and “Yes” to 2–4. Arrows indicate neutrophil clusters in wt. Scale bar = 100 μm. **e** Serum ALT levels. **f** Serum AST levels. **g** Serum albumin levels (*n* = 9–10/group). **h** Number of Ki67-positive proliferating cells in the liver sections. Arrows indicate Ki67-positive cells. Scale bar = 50 μm. **i** qPCR analysis of liver mRNA levels of gt/gt mice relative to wt, studied relative to TATA-box-binding protein mRNA. **a**, **b**, **e**–**i** Data are means ± SEM. *p < 0.05, **p < 0.01, ***p < 0.001. Alb, albumin; ALT, alanine aminotransferase; AST, aspartate aminotransferase; CHREBP, carbohydrate-responsive element-binding protein; GPX1, glutathione peroxidase 1; LPIN, lipin; MTTP, microsomal triglyceride transfer protein; PPARg2, peroxisome proliferator–activated receptor gamma 2; s, serum; TG, triglycerides

The liver weights of the *Hif-p4h-2*^*gt/gt*^ mice were > 20% lower than those of the WT after the HF-MCD diet (Fig. 5a). Consistently, the *Hif-p4h-2*^*gt/gt*^ livers had a significantly lower hepatic triglyceride content (Fig. 5b) and histological scores for steatosis severity, ~ 35% of the *Hif-p4h-2*^*gt/gt*^ livers against ~ 70% of WT scored as “very severe” (Fig. 5c). Only mild inflammation (Fig. 5d) and fibrosis (Fig. S5a) were detected to similar extents in both genotypes. Serum ALT and AST were elevated to a pathological level in both genotypes, but they were significantly ~ 30% lower in the *Hif-p4h-2*^*gt/gt*^ than in the WT mice (Fig. 5e, f). Additionally, the serum albumin levels were significantly higher for the *Hif-p4h-2*^*gt/gt*^ mice than for the WT (Fig. 5g), and the *Hif-p4h-2*^*gt/gt*^ livers had more proliferating cells than the WT (Fig. 5h) with no difference in apoptosis (Fig. S5b), suggesting enhanced regeneration in the *Hif-p4h-2*^*gt/gt*^ livers. Both genotypes’ livers showed similar dispersed expression of pericentral zonation marker glutamine synthetase (Fig. S5c). In agreement with less fat accumulation and damage detected in the *Hif-p4h-2*^*gt/gt*^ livers than in the WT, the hepatic expression levels of several lipid metabolism and oxidative stress mRNAs were lower, while the *Hif-p4h-2* mRNA downregulation was similar to that on the HFHF diet (Figs. 5i and S5d). Additionally, the levels of all cholesterol synthesis precursors but squalene were reduced in the *Hif-p4h-2*^*gt/gt*^ liver compared with that in the WT (Table S2), which suggests downregulated cholesterol synthesis in the former, likely contributing to the observed lower serum cholesterol levels (Fig. 4h). Additionally, serum uric acid levels were significantly lower in the *Hif-p4h-2*^*gt/gt*^ mice, while no differences were detected in the FGF21 levels (Fig. S5e, f). These data indicate that the *Hif-p4h-2*^*gt/gt*^ mice showed protection against the HF-MCD diet–induced hepatic fat accumulation and damage compared with the WT.

### Increased heat production in the HIF-P4H-2-deficient mice on an HF-MCD diet is associated with protection from NAFLD

**Fig. 6:**
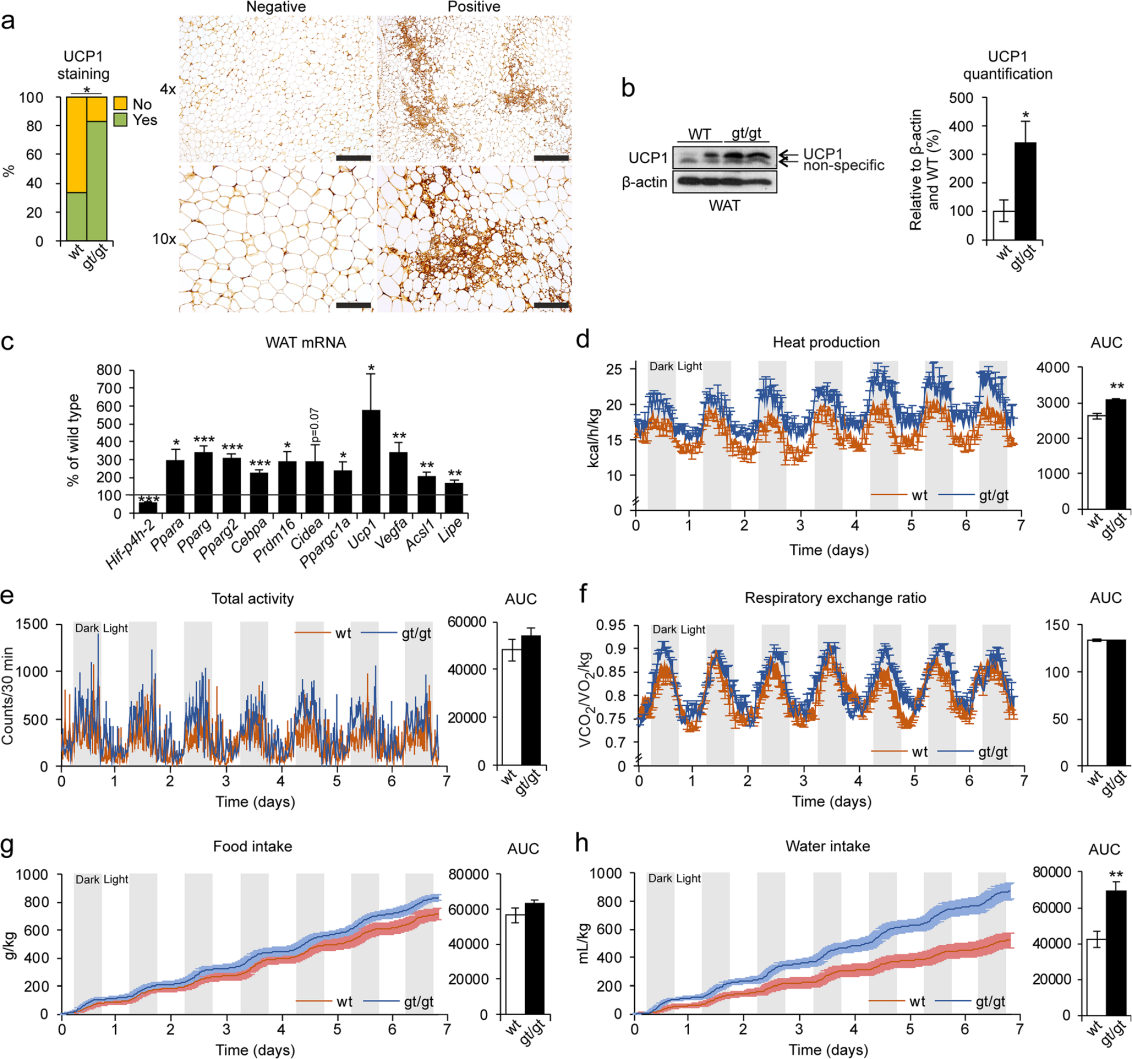
Heat production via UCP1 activation in the WAT is associated with protection from HF-MCD diet–induced NAFLD in HIF-P4H-2-deficient mice. Wild-type (wt) and Hif-p4h-2^gt/gt^ (gt/gt) females were studied while on a 7-week high-fat, methionine-choline-deficient (HF-MCD) diet. **a** Scoring of UCP1-stained WAT sections. “No” corresponds to < 5% of UCP1 staining/field and “Yes” to 5–25% (*n* = 10–12/group). Images are representative of scoring for wt. Scale bar = 500 μm for × 4 and 200 μm for × 10. **b** Western blotting and densitometric quantification of WAT UCP1 levels (*n* = 3–4/group.). β-Actin was used as a loading control. **c** qPCR analysis of the mRNA levels of browning markers in the WAT of the gt/gt mice relative to wt (*n* = 10–12/group). Gene expression was studied relative to β-actin mRNA. **d**–**h** Mice were analyzed in metabolic home cages for the last week of the diet (*n* = 7–8/group). **d** Heat production and AUC. **e** Total physical activity and AUC. **f** Respiratory exchange ratio (VCO_2_/VO_2_/kg) and AUC. **g** Food intake and AUC. **h** Water intake and AUC. **b**–**h** Data are means ± SEM. *p < 0.05, **p < 0.01, ***p < 0.001. ACSL1, acyl-CoA synthetase long-chain family member 1; AUC, area under the curve; CEBPA, CCAAT-enhancer-binding protein alpha; CIDEA, cell death–inducing DFFA-like effector A; LIPE, hormone-sensitive lipase; PPAR a/g, peroxisome proliferator–activated receptor alpha/gamma; PPARGC1A, PPARg coactivator-1 alpha; PRDM16, PR/SET domain-16; UCP1, uncoupling protein 1; VEGFA, vascular endothelial growth factor A; WAT, white adipose tissue

Browning of WAT has been shown to be a protective mechanism against NAFLD in mice on MCD diet [28, 29]. Therefore, we next studied the expression of uncoupling protein 1 (UCP1) in the WAT of the HF-MCD diet–fed mice. Surprisingly, the *Hif-p4h-2*^*gt/gt*^ mice showed significantly more UCP1 expression (83.3%) than the WT (33.3%) (Fig. 6a), verified by Western blotting (Fig. 6b). No difference between the genotypes in BAT UCP1 levels was detected (data not shown). In agreement, the mRNA levels of the browning markers *Ucp1*, *Ppara*, *Pparg*, *Pparg2*, *Cebpa*, *Prdm16*, *Vegfa*, *Ppargc1a*, *Acsl1*, and *Lipe* were significantly upregulated in the *Hif-p4h-2*^*gt/gt*^ WAT relative to the WT, the downregulation of *Hif-p4h-2* mRNA being similar to that on normal chow [14] (Fig. 6c). Since UCP1 expression relates to heat production, we submitted the mice on the HF-MCD diet for indirect gas calorimetry analysis, which showed significantly increased heat production by the *Hif-p4h-2*^*gt/gt*^ mice compared with the WT (Fig. 6d). There was no difference between the genotypes in physical activity, respiratory exchange ratio, or food intake (Fig. 6e–g), but an increase in water intake was observed in the *Hif-p4h-2*^*gt/gt*^ mice (Fig. 6h). These differences were not observed in previous measurements on normal chow [14], neither in the baseline measurements preceding the HF-MCD diet feeding, and no browning was detected in either genotype on the HFHF diet (data not shown). These data suggest that the *Hif-p4h-2*^*gt/gt*^ mice used advanced browning of WAT as a mechanism of protection against HF-MCD diet–induced NAFLD.

### Pharmacological inhibition of HIF-P4Hs ameliorates diet-induced obesity, metabolic dysfunction, and NAFLD

**Fig. 7:**
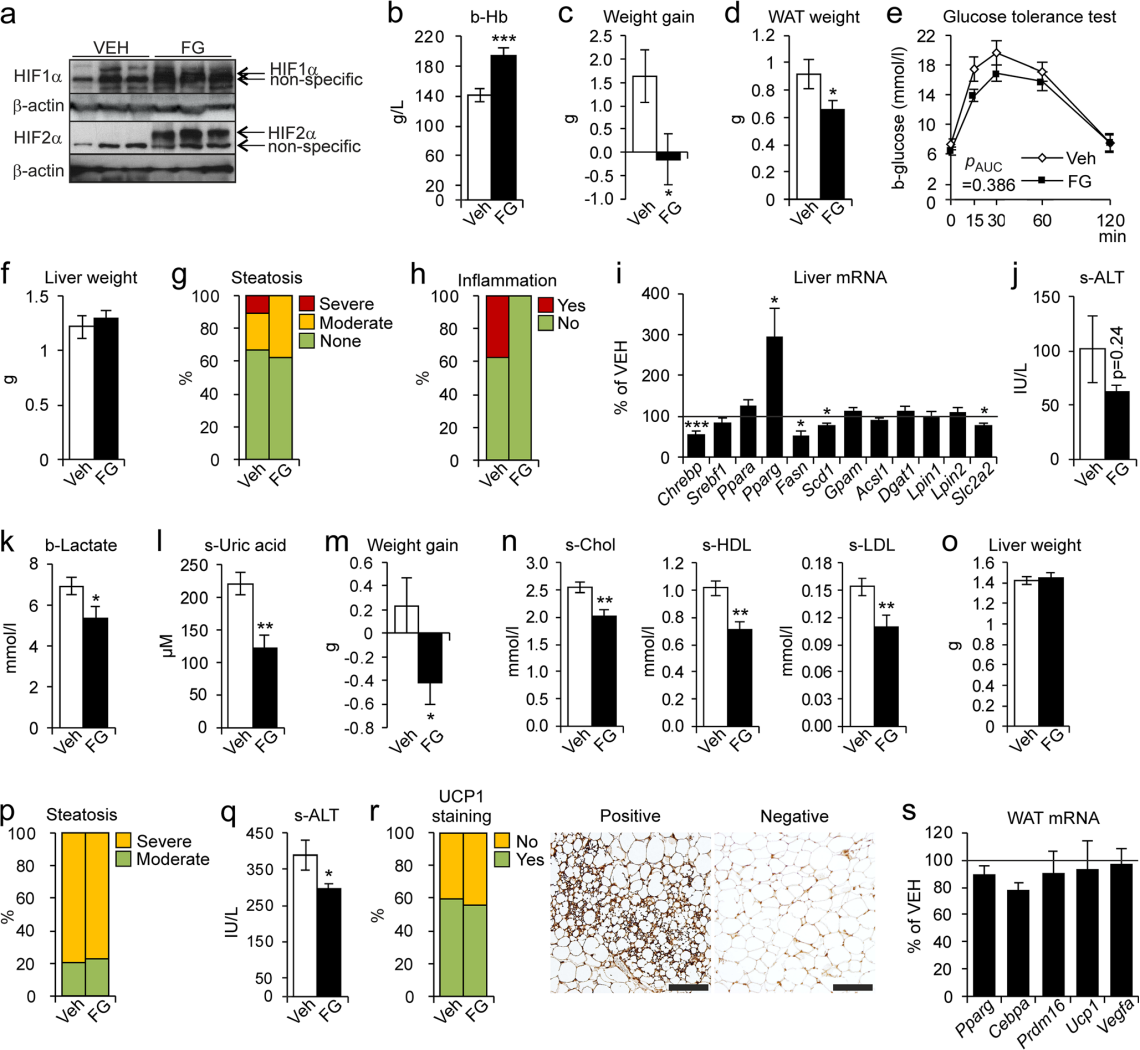
Pharmacological inhibition of HIF-P4Hs ameliorates diet-induced obesity, metabolic dysfunction, and liver damage. Wild-type (wt) females were fed a high-fat, high-fructose (HFHF) diet for **e** 4 or **a**–**d**, **f**–**l** 6 weeks or a high-fat, methionine-choline-deficient (HF-MCD) diet for **m**–**s** 3 weeks and simultaneously given vehicle (VEH) or 60 mg/kg of FG-4497 (FG) on days 1, 3, and 5 of each week (*n* = 8–10/group). **a** Western blot analysis of hepatic HIF1α and HIF2α protein levels. β-Actin was used as a loading control. **b** Blood hemoglobin levels. **c** Weight gain at the end of the diet relative to weights on the day before the diet started. **d** Weight of gonadal WAT. **e** Oral glucose tolerance test. The value for 0 min was determined after a 12-h fast. **f** Liver weight. **g** Scoring of hepatic steatosis. Steatosis grading: “None” corresponds to scores 0–2, “Moderate” to 3, and “Severe” to 4. **h** Scoring of liver inflammation. “No” corresponds to score 0 and “Yes” to 1–2. **i** qPCR analysis of liver mRNA levels of FG-treated mice relative to VEH-treated, studied relative to TATA-box-binding protein mRNA. **j** Serum ALT levels. **k** Blood lactate levels. **l** Serum uric acid levels. **m** Weight gain at the end of the HF-MCD diet relative to weights on the day before the diet started. **n** Serum total cholesterol, HDL cholesterol, and LDL cholesterol levels. **o** Liver weight. **p** Scoring of hepatic steatosis. Steatosis grading: “Moderate” corresponds to score 2 and “Severe” to 3. **q** Serum ALT levels. **r** Scoring of UCP1-stained WAT sections. “No” corresponds to < 5% of UCP1 staining/field and “Yes” to 5–25%. Images are representative of scoring for the VEH group. Scale bar = 200 μm. **s** qPCR analysis of WAT mRNA levels of FG-treated mice relative to VEH-treated group, studied relative to peptidylprolyl isomerase A mRNA. **b**–**f**, **i**–**o**, **q**, **s** Data are means ± SEM. *p ≤ 0.05, **p < 0.01, ***p < 0.001. ACSL1, acyl-CoA synthetase long-chain family member 1; ALT, alanine aminotransferase; b, blood; CEBPA, CCAAT-enhancer-binding protein alpha; CHREBP, carbohydrate-responsive element-binding protein; DGAT1, diacylglycerol o-acyltransferase 1; FASN, fatty acid synthase; GPAM, mitochondrial glycerol-3-phosphate acyltransferase; LPIN, lipin; PPAR a/g, peroxisome proliferator–activated receptor alpha/gamma; PRDM16, PR/SET domain-16; s, serum; SCD1, stearoyl-CoA desaturase 1; SLC2A2, solute carrier family-2 member 2; SREBF1c, sterol regulatory element-binding protein 1c; UCP-1, uncoupling protein 1; VEGFA, vascular endothelial growth factor A; WAT, white adipose tissue

WT mice were fed the HFHF diet for 6 weeks and simultaneously treated with a pharmacological pan-HIF-P4H inhibitor FG-4497 or vehicle. The FG-4497 treatment stabilized HIF1α and HIF2α in the livers (Fig. 7a) and elevated the blood hemoglobin levels (Fig. 7b). The FG-4497-treated mice did not gain weight (Fig. 7c) and had less WAT (Fig. 7d) than the vehicle-treated mice. They also showed a trend towards a better glucose tolerance (Fig. 7e). However, no significant differences in liver weight or steatosis were detected between groups (Fig. 7f, g), liver inflammation being detected only in the vehicle-treated group (Fig. 7h). The inability of the FG-4497 treatment to protect against steatosis compared with the *Hif-p4h-2*^*gt/gt*^ livers associated with only a few lipogenic mRNAs and *Slc2a2* being downregulated, and conversely with *Pparg* mRNA upregulation (Fig. 7i). Although not significant, the serum ALT levels were ~ 40% lower in the FG-4497 group (Fig. 7j), indicating less liver damage, possibly stemming from lower intake of fructose due to *Slc2a2* downregulation. Moreover, systemic levels of lactate and uric acid were significantly lower in the FG-4497-treated group (Fig. 7k, l). In conclusion, the FG-4497 treatment stabilized HIF, protecting from the HFHF diet–induced obesity and hepatic inflammation, but not from steatosis.

We next fed WT mice the HF-MCD diet for 3 weeks with simultaneous FG-4497 or vehicle treatment. The FG-4497 treatment was protected from weight gain (Fig. 7m) and the mice had a healthier serum lipid profile than the vehicle-treated group (Fig. 7n), but no difference in liver weight or steatosis was detected between the groups (Fig. 7o, p). Nevertheless, the FG-4497-treated mice had lower serum ALT levels (Fig. 7q). Lastly, similar expression levels of WAT UCP1 and browning marker mRNAs were detected in both groups (Fig. 7r, s). In conclusion, the FG-4497 treatment on the HF-MCD diet was protected from obesity, hypercholesterolemia, and liver damage, but could not induce browning of WAT to the extent seen in the *Hif-p4h-2*^*gt/gt*^ mice and thus, was unable to protect against steatosis.

## Discussion

Obesity and WAT function play key roles in the progression of NAFLD, since obesity and IR induce adipose tissue lipolysis and the liver then accumulates circulating FAs in a concentration-dependent manner [30]. Fructose contributes to NAFLD by providing a substrate for increased FA synthesis and activating hepatic de novo lipogenesis, which it promotes directly by upregulating SREBP1c [31], and indirectly by uric acid production, which reduces mitochondrial β-oxidation resulting in oxidative stress, hepatic IR, and endoplasmic reticulum (ER) stress [32, 33]. Absorbed fructose is transported via the portal vein, thus reaching the liver in higher concentrations than the other tissues.

The roles of HIFs and HIF-P4Hs in NAFLD are complex and not yet fully understood. The data available until this study is mainly from liver/hepatocyte-specific settings and suggest that stabilization of HIF2α, or simultaneous inhibition of all HIF-P4Hs, promotes NAFLD while restricting the inhibition to selected isoenzymes may have beneficial effects [14, 34–38]. We have reported earlier that normal chow-fed *Hif-p4h-2*^*gt/gt*^ mice have hepatic stabilization of HIF2α but not of HIF1α with upregulation of *Irs2* and downregulation of *Srebf1c* mRNA, resulting in lower acetyl-CoA levels, reduced de novo lipogenesis, and lower IR than the WT [14, 17]. Furthermore, we recently showed that mouse embryonic fibroblasts isolated from *Hif-p4h-2*^*gt/gt*^ mice show ~ 50% reduced ATP production compared with WT cells, likely resulting in the baseline differences in adiposity [14, 18]. We now show that *Hif-p4h-2*^*gt/gt*^ mice were able to resist weight gain and adiposity on a HFHF diet and had less hepatic steatosis and liver damage and no inflammation or fibrosis. These features associated with downregulation of lipogenic mRNAs, lower levels of hepatic acetyl-CoA and serum insulin, and better glucose tolerance. Moreover, differences in fructose metabolism in favor of the *Hif-p4h-2*^*gt/gt*^ mice were detected, including 20–33% less fructose found in the liver, kidney, and serum than in the WT, and lower levels of serum uric acid and lactate. Interestingly, the mRNA levels and enzymatic activity of KHKs, which are responsible for the cleavage of fructose, were upregulated in the small intestine of the *Hif-p4h-2*^*gt/gt*^ mice. The small intestine is the major site of fructose metabolism when ingested in low doses, whereas fructose spills over into the liver at high doses [26]. Our data suggest that inhibition of HIF-P4H-2 increases the capacity of the intestine to metabolize high doses of dietary fructose and therefore provides protection from its spillover into the liver and other tissues.

Moreover, the *Hif-p4h-2*^*gt/gt*^ mice on a HFHF diet expressed hepatic mRNAs for *Pnpla2*, *Pnpla3*, and *Cyp2e1* at lower levels than the WT. Inhibition of the adipose ligase PNPLA2 has been associated with protection against ER stress in mice [39, 40], while activating genetic variants in *PNPLA3* are associated with human NAFLD [41]. Moreover, increased expression of hepatic CYP2E1, which carries out the omega-hydroxylation of FAs, has been associated with NASH in mice and humans [42].

Additionally, the *Hif-p4h-2*^*gt/gt*^ mice on the HF-MCD diet had less WAT, and interestingly, they showed improved browning of WAT, which was associated with increased heat production. This likely contributed to less hepatic steatosis in the *Hif-p4h-2*^*gt/gt*^ mice, as WAT browning protecting against NAFLD has been reported on an MCD diet [28, 29]. However, this is the first report associating HIF-P4H inhibition with induced browning of WAT and increased thermogenesis, while earlier reports associate environmental hypoxia with HIF pathway activation and WAT browning [43, 44]. This likely involves the observed upregulation of adipose tissue PPARγ, which is a HIF target gene and essential in WAT browning [45–47].

Even though fructose is also required to develop liver injury on the HF-MCD diet, which contains sucrose [48], this diet additionally prevents hepatic VLDL export further compromising liver metabolism. This likely explains the lower degree of downregulation of hepatic lipid metabolism genes in the *Hif-p4h-2*^*gt/gt*^ mice on the HF-MCD diet compared with the HFHF diet.

NAFLD treatment is currently based on diet and exercise, while several therapeutics are being used with varying results. We show here that beneficial effects could be obtained with HIF-P4H-2 inhibition, as its genetic deficiency counteracted the symptoms of NAFLD. However, treatment with pan-HIF-P4H inhibitor was unable to reduce steatosis, as it demonstrated reduced ability to downregulate hepatic lipogenic gene expression in the HFHF diet and failure to significantly induce WAT browning in the HF-MCD diet. These data suggest that treatment of NAFLD by HIF-P4H inhibition should target only isoenzyme 2. This is supported by previous studies that associate the knockout of all HIF-P4Hs with steatosis, while the presence of a single HIF-P4H-1 or HIF-P4H-3 allele minimizes steatosis [49]. In conclusion, our findings demonstrate important underlying mechanisms of HIF-P4H-2 interference involving organ crosstalk in the pathogenesis of NAFLD, which offers potential for developing HIF-P4H-2 inhibitors for its treatment.

## Electronic supplementary material

ESM 1 (PDF 847 kb)

## References

[1] Diehl AM, Day C (2017). Cause, pathogenesis, and treatment of nonalcoholic steatohepatitis. N Engl J Med.

[2] Asrani SK, Devarbhavi H, Eaton J, Kamath PS (2019). Burden of liver diseases in the world. J Hepatol.

[3] Asrih M, Jornayvaz FR (2014). Diets and nonalcoholic fatty liver disease: the good and the bad. Clin Nutr.

[4] Leoni S, Tovoli F, Napoli L, Serio I, Ferri S, Bolondi L (2018). Current guidelines for the management of non-alcoholic fatty liver disease: a systematic review with comparative analysis. World J Gastroenterol.

[5] Kaelin WG, Ratcliffe PJ (2008). Oxygen sensing by metazoans: the central role of the HIF hydroxylase pathway. Mol Cell.

[6] Koivunen P, Serpi R, Dimova EY (2016). Hypoxia-inducible factor prolyl 4-hydroxylase inhibition in cardiometabolic diseases. Pharmacol Res.

[7] Semenza GL (2012). Hypoxia-inducible factors in physiology and medicine. Cell.

[8] Berra E, Benizri E, Ginouves A, Volmat V, Roux D, Pouyssegur J (2003). HIF prolyl-hydroxylase 2 is the key oxygen sensor setting low steady-state levels of HIF-1alpha in normoxia. EMBO J.

[9] Hirsilä M, Koivunen P, Günzler V, Kivirikko KI, Myllyharju J (2003). Characterization of the human prolyl 4-hydroxylases that modify the hypoxia-inducible factor. J Biol Chem.

[10] Koivunen P, Kietzmann T (2018). Hypoxia-inducible factor prolyl 4-hydroxylases and metabolism. Trends Mol Med.

[11] Dhillon S (2019) Roxadustat: first global approval. Drugs 10.1007/s40265-019-01077-1 30805897

[12] Matsuura H, Ichiki T, Inoue E, Nomura M, Miyazaki R, Hashimoto T, Ikeda J, Takayanagi R, Fong G, Sunagawa K (2013). Prolyl hydroxylase domain protein 2 plays a critical role in diet-induced obesity and glucose intolerance. Circulation.

[13] Marsch E, Demandt JAF, Theelen TL, Tullemans BME, Wouters K, Boon MR, van Dijk TH, Gijbels MJ, Dubois LJ, Meex SJR, Mazzone M, Hung G, Fisher EA, Biessen EA, Daemen MJ, Rensen PC, Carmeliet P, Groen AK, Sluimer JC (2016). Deficiency of the oxygen sensor prolyl hydroxylase 1 attenuates hypercholesterolaemia, atherosclerosis, and hyperglycaemia. Eur Heart J.

[14] Rahtu-Korpela L, Karsikas S, Horkko S, Blanco Sequeiros R, Lammentausta E, Makela KA, Herzig KH, Walkinshaw G, Kivirikko KI, Myllyharju J (2014). HIF prolyl 4-hydroxylase-2 inhibition improves glucose and lipid metabolism and protects against obesity and metabolic dysfunction. Diabetes.

[15] Voss JD, Masuoka P, Webber BJ, Scher AI, Atkinson RL (2013). Association of elevation, urbanization and ambient temperature with obesity prevalence in the United States. Int J Obes.

[16] Woolcott OO, Ader M, Bergman RN (2015). Glucose homeostasis during short-term and prolonged exposure to high altitudes. Endocr Rev.

[17] Rahtu-Korpela L, Maatta J, Dimova EY, Horkko S, Gylling H, Walkinshaw G, Hakkola J, Kivirikko KI, Myllyharju J, Serpi R (2016). Hypoxia-inducible factor prolyl 4-hydroxylase-2 inhibition protects against development of atherosclerosis. Arterioscler Thromb Vasc Biol.

[18] Laitakari A, Huttunen R, Kuvaja P, Hannuksela P, Szabo Z, Heikkilä M, Kerkelä R, Myllyharju J, Dimova EY, Serpi R et al (2020) Systemic long-term inactivation of hypoxia-inducible factor prolyl 4-hydroxylase 2 ameliorates aging-induced changes in mice without affecting their life span. FASEB J 10.1096/fj.201902331R 32100354

[19] Laitakari A, Ollonen T, Kietzmann T, Walkinshaw G, Mennerich D, Izzi V, Haapasaari K, Myllyharju J, Serpi R, Dimova EY (2019). Systemic inactivation of hypoxia-inducible factor prolyl 4-hydroxylase 2 in mice protects from alcohol-induced fatty liver disease. Redox Biol.

[20] Ganz M, Bukong TN, Csak T, Saha B, Park J, Ambade A, Kodys K, Szabo G (2015). Progression of non-alcoholic steatosis to steatohepatitis and fibrosis parallels cumulative accumulation of danger signals that promote inflammation and liver tumors in a high fat-cholesterol-sugar diet model in mice. J Transl Med.

[21] Matsumoto M, Hada N, Sakamaki Y, Uno A, Shiga T, Tanaka C, Ito T, Katsume A, Sudoh M (2013). An improved mouse model that rapidly develops fibrosis in non-alcoholic steatohepatitis. Int J Exp Pathol.

[22] Caballero F, Fernandez A, Matias N, Martinez L, Fucho R, Elena M, Caballeria J, Morales A, Fernandez-Checa JC, Garcia-Ruiz C (2010). Specific contribution of methionine and choline in nutritional nonalcoholic steatohepatitis: impact on mitochondrial S-adenosyl-L-methionine and glutathione. J Biol Chem.

[23] Hyvarinen J, Hassinen IE, Sormunen R, Maki JM, Kivirikko KI, Koivunen P, Myllyharju J (2010). Hearts of hypoxia-inducible factor prolyl 4-hydroxylase-2 hypomorphic mice show protection against acute ischemia-reperfusion injury. J Biol Chem.

[24] Merry TL, Tran M, Stathopoulos M, Wiede F, Fam BC, Dodd GT, Clarke I, Watt MJ, Andrikopoulos S, Tiganis T (2014). High-fat-fed obese glutathione peroxidase 1-deficient mice exhibit defective insulin secretion but protection from hepatic steatosis and liver damage. Antioxid Redox Signal.

[25] Merry TL, Tran M, Dodd GT, Mangiafico SP, Wiede F, Kaur S, McLean CL, Andrikopoulos S, Tiganis T (2016). Hepatocyte glutathione peroxidase-1 deficiency improves hepatic glucose metabolism and decreases steatohepatitis in mice. Diabetologia.

[26] Jang C, Hui S, Lu W, Cowan AJ, Morscher RJ, Lee G, Liu W, Tesz GJ, Birnbaum MJ, Rabinowitz JD (2018). The small intestine converts dietary fructose into glucose and organic acids. Cell Metab.

[27] Karsikas S, Myllymäki M, Heikkilä M, Sormunen R, Kivirikko KI, Myllyharju J, Serpi R, Koivunen P (2016). HIF-P4H-2 deficiency protects against skeletal muscle ischemia-reperfusion injury. J Mol Med.

[28] Jha P, Knopf A, Koefeler H, Mueller M, Lackner C, Hoefler G, Claudel T, Trauner M (2014). Role of adipose tissue in methionine-choline-deficient model of non-alcoholic steatohepatitis (NASH). Biochim Biophys Acta.

[29] Lee YH, Kim SH, Kim SN, Kwon HJ, Kim JD, Oh JY, Jung YS (2016). Sex-specific metabolic interactions between liver and adipose tissue in MCD diet-induced non-alcoholic fatty liver disease. Oncotarget.

[30] Guilherme A, Virbasius JV, Puri V, Czech MP (2008). Adipocyte dysfunctions linking obesity to insulin resistance and type 2 diabetes. Nat Rev Mol Cell Biol.

[31] Miyazaki M, Dobrzyn A, Man WC, Chu K, Sampath H, Kim H, Ntambi JM (2004). Stearoyl-CoA desaturase 1 gene expression is necessary for fructose-mediated induction of lipogenic gene expression by sterol regulatory element-binding protein-1c-dependent and -independent mechanisms. J Biol Chem.

[32] Lim JS, Mietus-Snyder M, Valente A, Schwarz J, Lustig RH (2010). The role of fructose in the pathogenesis of NAFLD and the metabolic syndrome. Nat Rev Gastroenterol Hepatol.

[33] Jensen T, Abdelmalek MF, Sullivan S, Nadeau KJ, Green M, Roncal C, Nakagawa T, Kuwabara M, Sato Y, Kang D, Tolan DR, Sanchez-Lozada LG, Rosen HR, Lanaspa MA, Diehl AM, Johnson RJ (2018). Fructose and sugar: a major mediator of non-alcoholic fatty liver disease. J Hepatol.

[34] Taniguchi CM, Finger EC, Krieg AJ, Wu C, Diep AN, LaGory EL, Wei K, McGinnis LM, Yuan J, Kuo CJ, Giaccia AJ (2013). Cross-talk between hypoxia and insulin signaling through Phd3 regulates hepatic glucose and lipid metabolism and ameliorates diabetes. Nat Med.

[35] Minamishima YA, Kaelin WG (2010). Reactivation of hepatic EPO synthesis in mice after PHD loss. Science.

[36] Morello E, Sutti S, Foglia B, Novo E, Cannito S, Bocca C, Rajsky M, Bruzzì S, Abate ML, Rosso C, Bozzola C, David E, Bugianesi E, Albano E, Parola M (2018). Hypoxia-inducible factor 2α drives nonalcoholic fatty liver progression by triggering hepatocyte release of histidine-rich glycoprotein. Hepatology.

[37] Rankin EB, Rha J, Selak MA, Unger TL, Keith B, Liu Q, Haase VH (2009). Hypoxia-inducible factor 2 regulates hepatic lipid metabolism. Mol Cell Biol.

[38] Kim WY, Safran M, Buckley MRM, Ebert BL, Glickman J, Bosenberg M, Regan M, Kaelin WG (2006). Failure to prolyl hydroxylate hypoxia-inducible factor alpha phenocopies VHL inactivation in vivo. EMBO J.

[39] Fuchs CD, Claudel T, Kumari P, Haemmerle G, Pollheimer MJ, Stojakovic T, Scharnagl H, Halilbasic E, Gumhold J, Silbert D, Koefeler H, Trauner M (2012). Absence of adipose triglyceride lipase protects from hepatic endoplasmic reticulum stress in mice. Hepatology.

[40] Schweiger M, Romauch M, Schreiber R, Grabner GF, Hütter S, Kotzbeck P, Benedikt P, Eichmann TO, Yamada S, Knittelfelder O (2017). Pharmacological inhibition of adipose triglyceride lipase corrects high-fat diet-induced insulin resistance and hepatosteatosis in mice. Nat Commun.

[41] Huang Y, Cohen JC, Hobbs HH (2011). Expression and characterization of a PNPLA3 protein isoform (I148M) associated with nonalcoholic fatty liver disease. J Biol Chem.

[42] Leung T, Nieto N (2013). CYP2E1 and oxidant stress in alcoholic and non-alcoholic fatty liver disease. J Hepatol.

[43] Gozal D, Gileles-Hillel A, Cortese R, Li Y, Almendros I, Qiao Z, Khalyfa AA, Andrade J, Khalyfa A (2017). Visceral white adipose tissue after chronic intermittent and sustained hypoxia in mice. Am J Respir Cell Mol Biol.

[44] van den Borst B, Schols AMWJ, de Theije C, Boots AW, Köhler SE, Goossens GH, Gosker HR (2013). Characterization of the inflammatory and metabolic profile of adipose tissue in a mouse model of chronic hypoxia. J Appl Physiol.

[45] Krishnan J, Suter M, Windak R, Krebs T, Felley A, Montessuit C, Tokarska-Schlattner M, Aasum E, Bogdanova A, Perriard E, Perriard JC, Larsen T, Pedrazzini T, Krek W (2009). Activation of a HIF1alpha-PPARgamma axis underlies the integration of glycolytic and lipid anabolic pathways in pathologic cardiac hypertrophy. Cell Metab.

[46] Vernochet C, Peres SB, Davis KE, McDonald ME, Qiang L, Wang H, Scherer PE, Farmer SR (2009). C/EBPalpha and the corepressors CtBP1 and CtBP2 regulate repression of select visceral white adipose genes during induction of the brown phenotype in white adipocytes by peroxisome proliferator-activated receptor gamma agonists. Mol Cell Biol.

[47] Ohno H, Shinoda K, Spiegelman BM, Kajimura S (2012). PPARγ agonists induce a white-to-brown fat conversion through stabilization of PRDM16 protein. Cell Metab.

[48] Pickens MK, Yan JS, Ng RK, Ogata H, Grenert JP, Beysen C, Turner SM, Maher JJ (2009). Dietary sucrose is essential to the development of liver injury in the methionine-choline-deficient model of steatohepatitis. J Lipid Res.

[49] Duan L, Takeda K, Fong G (2014). Hematological, hepatic, and retinal phenotypes in mice deficient for prolyl hydroxylase domain proteins in the liver. Am J Pathol.

