# Supplementary material for: HIF-P4H-2 inhibition enhances intestinal fructose metabolism and induces thermogenesis protecting against NAFLD
Source: J Mol Med (Berl). 2020 Apr 15;98(5):719–31. doi: 10.1007/s00109-020-01903-0 (PMC7220983; doi:10.1007/s00109-020-01903-0)
Supplement: Supplementary file 1 — (PDF 847 kb) [file 109_2020_1903_MOESM1_ESM.pdf]

HIF-P4H-2 inhibition enhances intestinal fructose metabolism and induces thermogenesis protecting against NAFLD

Anna Laitakari[1], Joona Tapio[1], Kari A. Mäkelä[2], Karl-Heinz Herzig[2,3], Franziska Dengler[4], Helena Gylling[5], Gail Walkinshaw[6], Johanna Myllyharju[1], Elitsa Y. Dimova[1], Raisa Serpi[1], Peppi Koivunen[1,*]

[1]Biocenter Oulu, Faculty of Biochemistry and Molecular Medicine, Oulu Center for Cell-Matrix Research, University of Oulu, Oulu, Finland. [2]Research Unit of Biomedicine, Biocenter Oulu, Medical Research Center and University Hospital, Oulu, Finland. [3]Department of Gastroenterology and Metabolism, Poznan University of Medical Sciences, Poznan, Poland. [4]University of Leipzig, Leipzig, Germany. [5]University of Helsinki and Helsinki University Hospital, Internal Medicine, 00029 HUS, Helsinki, Finland. [6]FibroGen, Inc., San Francisco, CA, USA.

Contact information

[*]Corresponding author. Address: Faculty of Biochemistry and Molecular Medicine, University of Oulu, Aapistie 7C, FIN-90014 Oulu, Finland. Tel.: +358 294 485822. E-mail address: peppi.koivunen@oulu.fi. ORCID ID: 0000-0002-2827-8229

Content:

Supplementary materials and methods, supplementary figures (5) and supplementary tables (2).

Supplementary materials and methods

Animal experiments

Power calculations were performed prior to the experiments in order to determine the smallest number of animals needed for significant data. Animals were housed in the Laboratory Animal Center of the University of Oulu on a 12 h light/dark cycle, at a constant temperature of 21-22°C in groups of 2-6. The well-being of the mice was monitored daily and their body weight measured once per week in the genetic models and three times per week in the pharmacological models. FG-4497 used in pharmacologic studies was dissolved in 0.5% sodium carboxymethyl cellulose and 0.1% polysorbate-80, also used as a vehicle. For the fructose diet 5-month-old *Hif-p4h-2*$^{gt/gt}$ females were fed a 30% (w/v) fructose solution for drinking-water with a standard rodent diet (Teklad 2018, Envigo) for 16 weeks. The special diets were from Research Diets (USA).

Fructose intake

Intake of 30% fructose solution was determined daily by measuring the weight of the solution left.

^{14}C-fructose uptake

5-6-month-old female mice were fed the HFHF diet for 2 weeks. After a 12 h fast, the mice were orally gavaged with 0.3 µCi/g ^{14}C-fructose (MOR-MC 1459-250, Moravek) combined with 3 mg/g fructose and sacrificed at 15 min. Tissue samples were homogenized with chloroform:methanol and centrifuged. The supernatant and serum were scintillated for ^{14}C activity.

Fructose secretion measurements

To measure the secretion of ^{14}C-fructose, 2.5-month-old female mice were subjected to a 6 h fast, after which they were orally gavaged with 0.3 µCi/g ^{14}C-fructose (MOR-MC 1459-250, Moravek)

combined with 3 mg/g fructose. Feces and urine were collected for 24 h and 2 h, respectively, and 50 µl samples of urine and the measured feces were scintillated for ^{14}C activity.

Ketohexokinase (KHK) activity

KHK activity was measured in female mice after 4 weeks on the HFHF diet, as described by others [1,2]. In brief, 125 ug of jejunum protein was incubated at +37°C in a reaction mixture. Fructose in the supernatant was detected according to Roe [3] and absorbance read at 515 nm. Reactions without NaATP and $MgCl_2$ were used as negative controls.

Blood and serum analyses

The serum alanine aminotransferase (ALT), aspartate aminotransferase (AST), albumin, uric acid, total cholesterol, HDL, LDL and triglycerides were determined by clinical analytical methods (Nordlab, Oulu University Hospital). FFA Quantification Kit (ab65341, Abcam) and Mouse Leptin ELISA (EZML-82K, Millipore) were used to determine their serum levels and ALT activity assay (MAK052, Sigma-Aldrich) the ALT-levels of the pharmacological models. Levels of circulating fibroblast growth factor 21 (FGF21) were determined with the Mouse/Rat FGF-21 Quantikine ELISA Kit (MF2100, R&D Systems). A lactometer (SensLab/EKF Diagnostics) and a hemoglobin meter (Triolab) were used for the corresponding analyses. Glucose tolerance test (GTT) was performed and serum insulin levels and HOMA-IR were determined as previously described [4], 2 weeks before sacrifice.

Hepatic triglycerides and acetyl-CoA

Hepatic triglyceride and acetyl-CoA concentrations were determined as previously described [4,5].

Determination of cholesterol synthesis and absorption biomarkers in the liver

Liver cholesterol, squalene, lanosterol, desmosterol, cholestenol, lathosterol, cholestanol, campesterol and sitosterol were quantified by capillary gas-liquid chromatography (GLC, Agilent 6890N Network GC System, Agilent Technologies) using a 50 m non-polar Ultra 2 capillary column (5 % phenyl-methyl siloxane, Agilent Technologies) with 5α-cholestane as an internal standard.

Histological analyses

Formalin-fixed, paraffin-embedded liver and gonadal white adipose tissue (WAT) sections were stained with hematoxylin-eosin (H&E). Hepatic steatosis and inflammation (neutrophils) from H&E-stained and fibrosis from Masson's Trichrome-stained sections were scored (0-4). Areas of 100 adipocytes were quantified from H&E-stained WAT sections with Adobe Photoshop CS6, CD68-positive macrophages in WAT quantified with an anti-CD68 antibody (ab955, Abcam) and hepatic proliferating cells with an anti-Ki67 antibody (NCL-Ki67-MM1, Leica Biosystems) from five 20x fields/mouse. WAT browning was scored (0-4) with an anti-UCP1 antibody (U6382, Sigma-Aldrich).

Liver fibrosis for the HF-MCD model was determined from Masson's Trichrome stained tissue sections as the percentage of positively stained area from all tissue area with the NIS-Elements BR software (Nikon). Five hot-spot high-power-field images were evaluated from each sample. Liver apoptosis was analysed from liver sections stained with the *In Situ* Cell Death Detection kit, fluorescein (11684795910, Roche), and the number of apoptotic cells was calculated from 5 fields per mouse. Liver pericentral zonation was evaluated with an anti-glutamine synthetase (GS) antibody (610518, BD Biosciences).

Intestinal GLUT2 expression was determined with primary anti-GLUT2 (sc-7680, Santa Cruz Biotechnology) and secondary (Cy3, 705-165-147, Dianova, Hamburg, Germany) antibodies and the nuclei were stained with DAPI (Carl Roth, Karlsruhe, Germany). The staining was assessed using a Leica TCS SP8 confocal laser scanning microscope and the Leica Application Suite X (LAS-X)

software (Leica, Wetzlar, Germany). Each section was exposed for an overview scan, where five positions with distinguishable photon counts in the epithelial structures were selected and scanned, and the brush border membrane and the nuclei were marked. The latter were used to normalize the former in order to adjust for differences in total staining intensities. For each area, ten regions of interest (2 x 2 µm) were positioned and the number of photons counted in each region.

Quantitative real-time PCR (qPCR)

RNA was isolated and qPCR analyses performed as previously described [5] using primers shown in Table S1.

Western blot

Proteins were extracted (NE-PER kit, Pierce) and immunoblotting performed with antibodies against HIF1α, HIF2α, β-actin (NB-100-479, NB100-122, NB600-501, all Novus Biologicals), FAS (C20G5, Cell Signaling Technology), UCP1 (U6382, Sigma-Aldrich) and α-tubulin (T6199, Sigma-Aldrich). Densities of bands were quantified with Fiji (ImageJ) and normalized to α-tubulin or β-actin.

Primary hepatocytes were isolated from age-matched WT and *Hif-p4h-2*$^{gt/gt}$ mice fed normal chow by a standard two-step non-recirculating perfusion via the *vena cava*. For protein isolation the hepatocytes were lysed in a buffer (50 mM Tris–HCl, pH 8, 150 mM NaCl, 0.5% NP-40, 1 mM PMSF) and centrifuged. 100 µg of proteins were resolved by SDS-PAGE, blotted, and probed with the following primary antibodies: HIF-P4H-2 (NB100-2219, Novus Biologicals) and vinculin (sc-5573, Santa Cruz). The Pierce ECL system (ThermoScientific) was used for detection. Densities of HIF-P4H-2 bands were quantified with Fiji (ImageJ) and levels normalized to vinculin levels.

Statistics

Student's two-tailed *t*-test was used to compare two groups and Fisher's exact test for histological scoring data. Data are presented as means ± SEM unless otherwise stated. $p<0.05$ was considered statistically significant.

Supplementary figures

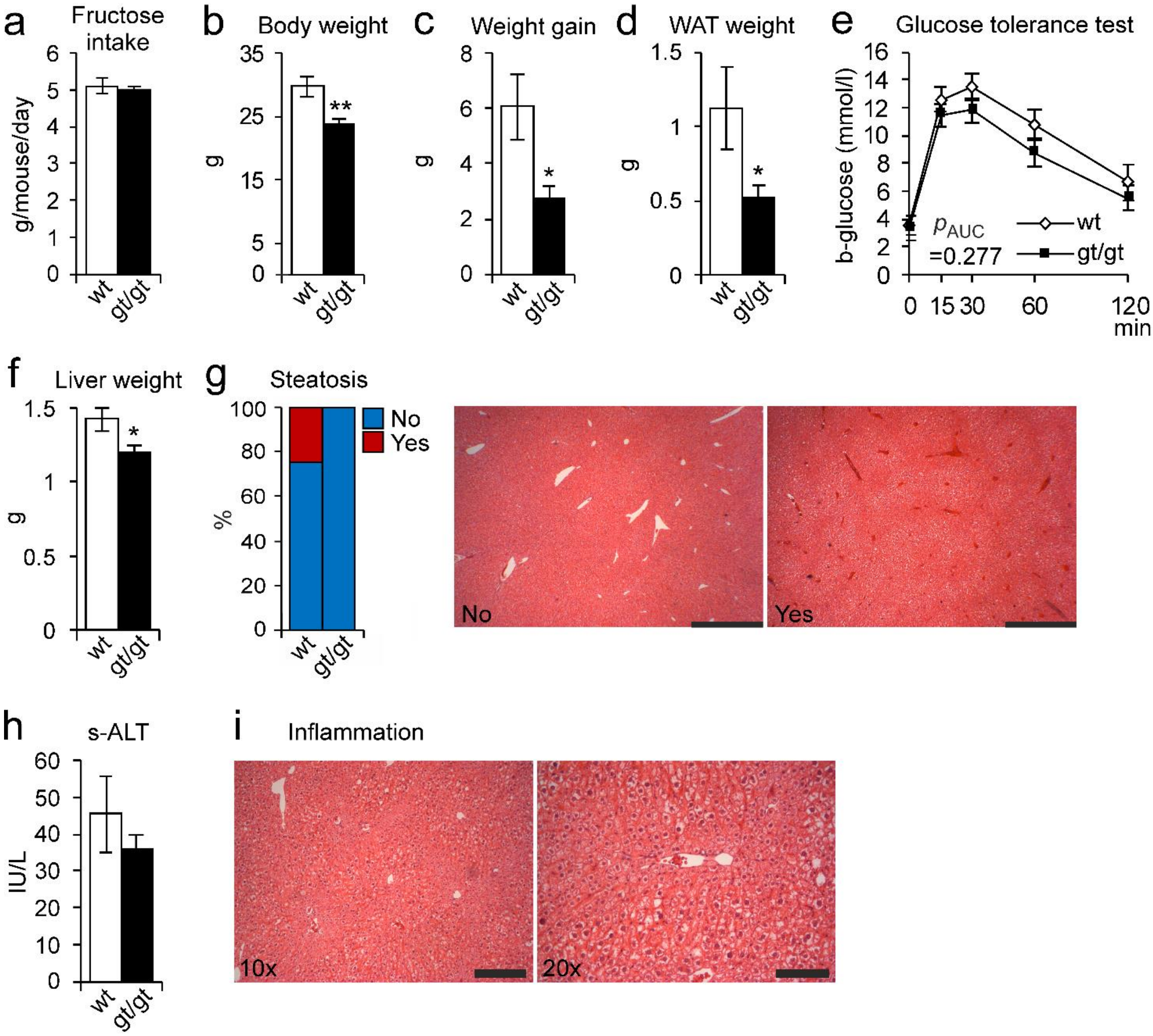

Fig. S1. HIF-P4H-2-deficient mice are protected against high-fructose diet-induced obesity and increased liver weight. Wild-type (wt) and *Hif-p4h-2*$^{gt/gt}$ (gt/gt) females were studied while on a 16-week standard rodent diet with 30% fructose solution as drinking water (n=8-10/group). (g, i) Images are representative of scoring for wt. (a) Fructose solution intake. (b) Body weight at sacrifice. (c) Weight gain at the end of the diet relative to weights on the day before the diet started. (d) Weight of gonadal WAT. (e) Oral glucose tolerance test performed at 13.5 weeks of the diet. The value for 0 minutes was determined after a 12 h fast. (f) Liver weight. (g) Scoring of steatosis and H&E-stained liver sections. For steatosis grading “No” corresponds to scores 0-2 and “Yes” to 3-4. Scale bar=500 µm. (h) Serum ALT levels. (i) Liver inflammation in H&E-stained liver sections. Scale bar=200 µm for 10x and 100 µm for 20x. (a-f, h) Data are means ± SEM. *p <0.05, **p <0.01. Abbreviations: ALT, alanine aminotransferase; s, serum; WAT, white adipose tissue

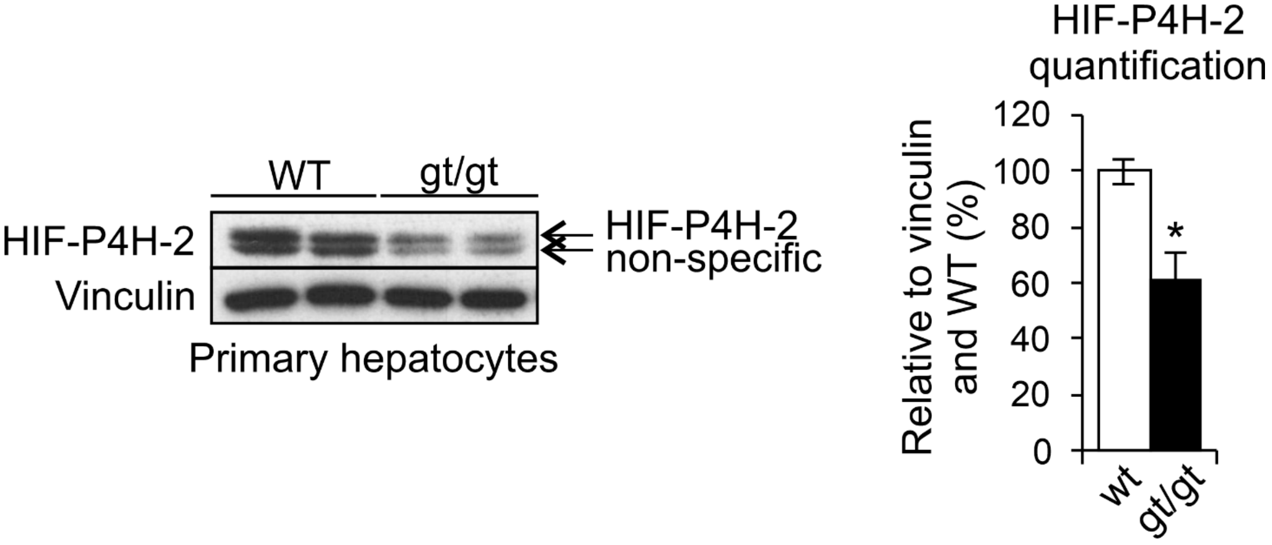

Fig. S2. Western blot analysis and densitometric quantification of hepatic HIF-P4H-2 protein levels. Primary hepatocytes were isolated from wild-type (WT) and *Hif-p4h-2*$^{gt/gt}$ (gt/gt) mice (n=3-4/group). Vinculin was used as a loading control. Data are means ± SEM. *p <0.05.

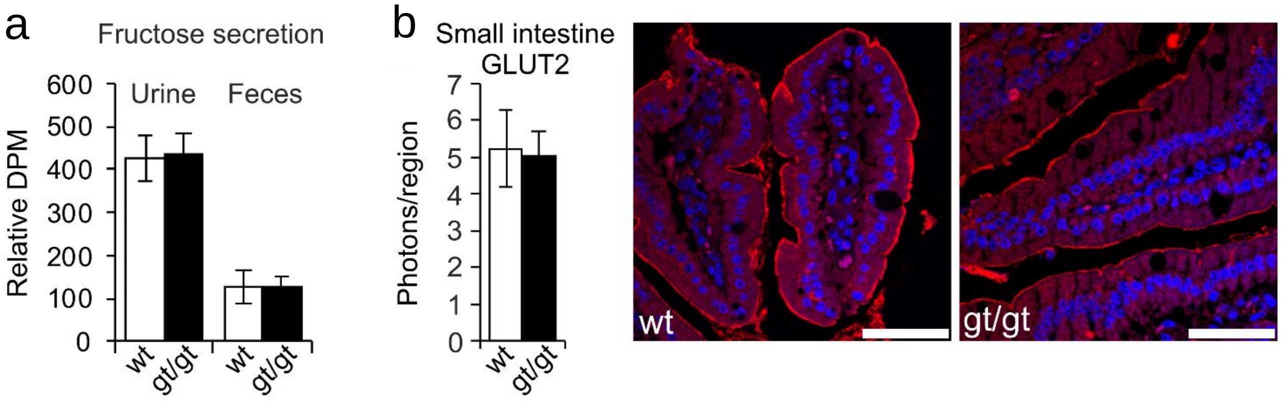

Fig. S3. ^{14}C-fructose secretion and intestinal GLUT2 expression. (a) After 2 weeks on the high-fat, high-fructose (HFHF) diet, and following a 6 h fast, wild-type (wt) and *Hif-p4h-2$^{gt/gt}$* (gt/gt) mice received ^{14}C-fructose orally and its secretion in urine in 2 h (n=5/group) and feces in 24 h (n=7/group) was determined. The samples were measured for ^{14}C radioactivity as DPM/mg. (b) GLUT2 protein expression in the small intestine of the wt and *Hif-p4h-2$^{gt/gt}$* mice after an 8-week HFHF diet (n=8-10/group). Scale bar=50 μm. Data are means ± SEM. Abbreviations: DPM, disintegration per minute; GLUT2, glucose transporter 2

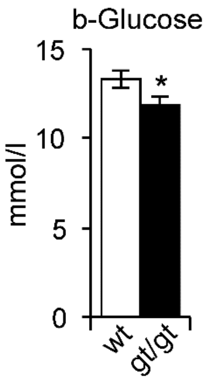

Fig. S4. HIF-P4H-2-deficient mice have lower blood glucose than the WT on the HF-MCD diet. Wild-type (wt) and *Hif-p4h-2*$^{gt/gt}$ (gt/gt) mice after 7 weeks on the high-fat, methionine-choline-deficient (HF-MCD) diet (n=9-12/group). Data are means ± SEM. **p* <0.05. Abbreviations: b, blood

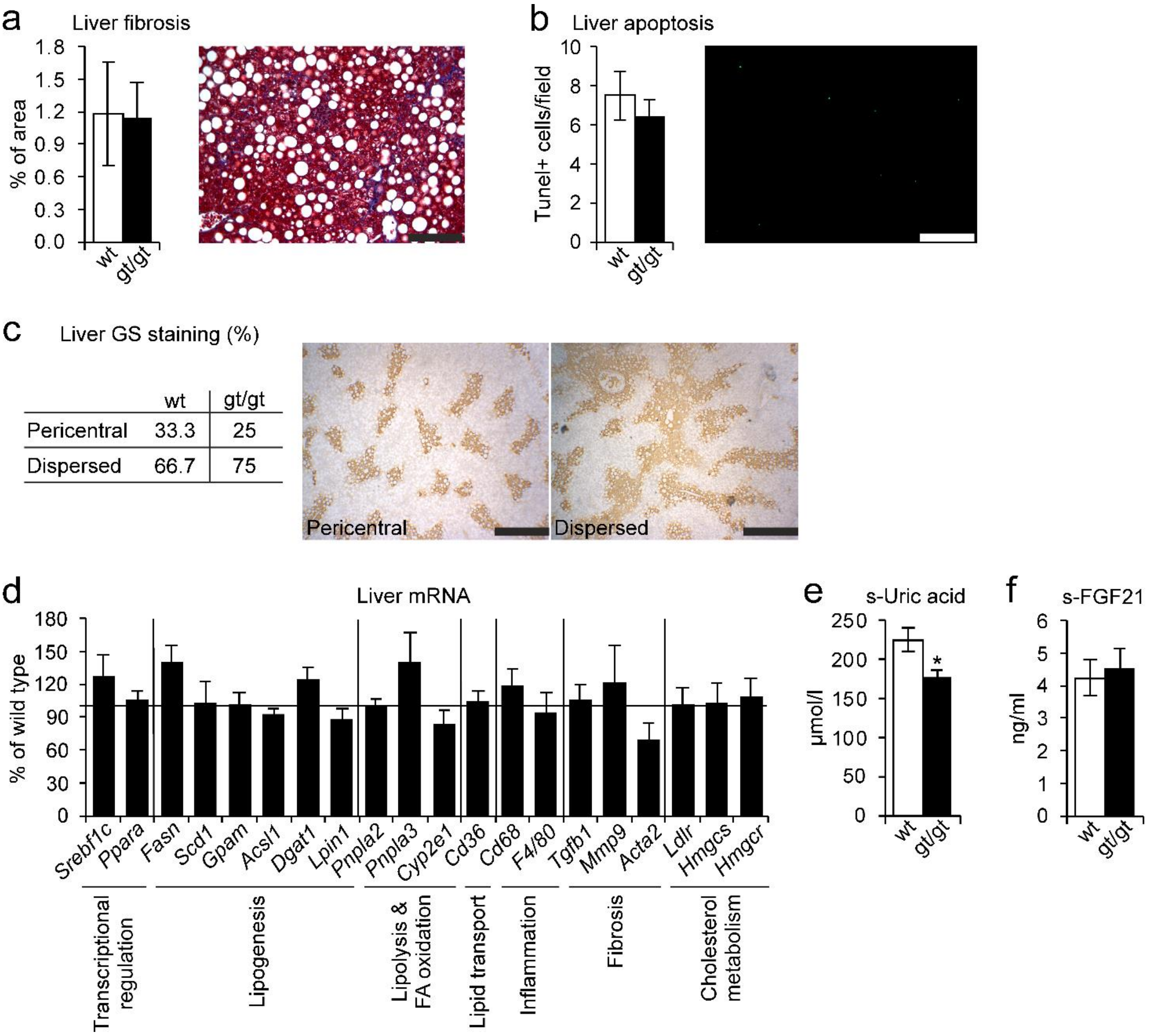

Fig. S5. Analyses of liver histology, mRNA levels and serum markers in the HIF-P4H-2-deficient and wt mice on the HF-MCD diet. Wild-type (wt) and *Hif-p4h-2*$^{gt/gt}$ (gt/gt) mice after 7 weeks on the high-fat, methionine-choline-deficient (HF-MCD) diet (n=10-12/group). (a-c) Images are representative of scoring for wt. (a) Quantification of fibrosis from Masson's trichrome-stained liver sections. Scale bar=100 µm. (b) Analysis of Tunel-positive apoptotic cells per field in liver sections. Scale bar=100 µm. (c) Scoring for GS-staining from liver sections showing liver pericentral zones. Scale bar=500 µm. (d) qPCR analysis of liver mRNA levels of gt/gt mice relative to wt, studied relative to TATA-box binding protein mRNA. (e) Serum uric acid levels. (f) Serum FGF21 levels. (a, b, d-f): Data are means ± SEM. *p <0.05. Abbreviations: ACSL1, acyl-CoA synthetase long chain family member-1; ACTA2, actin alpha 2, smooth muscle; CYP2E1, cytochrome P450 family-2 subfamily e member-1; DGAT1, diacylglycerol o-acyltransferase-1; FA, fatty acid; FASN, fatty acid synthase; FGF21, fibroblast growth factor 21; GPAM, mitochondrial glycerol-3-phosphate acyltransferase; GPX1, glutathione peroxidase-1; GS, glutamine synthetase; HMGCS/HMGCR,

hydroxymethylglutaryl-CoA synthase/reductase; LDLR, low density lipoprotein receptor; LPIN, lipin; MMP9, matrix metallopeptidase 9; PNPLA, patatin like phospholipase domain containing; PPARα, peroxisome proliferator-activated receptor alpha; s, serum; SCD1, stearoyl-CoA desaturase-1; SREBF1c, sterol regulatory element-binding protein 1c; TGFb1, transforming growth factor beta 1

Supplementary tables

Supplementary Table 1. Primers used in the quantitative PCR analyses.

Gene	Forward primer (5'→3')	Reverse primer (5'→3')
Acta2	GTCCCAGACATCAGGGAGTAA	TCGGATACTTCAGCGTCAGGA
Actb	AGAGGGAAATCGTGCGTGAC	CAATAGTGATGACCTGGCCGT
Acsl1	TGCCAGAGCTGATTGACATTC	GGCATACCAGAAGGTGGTGAG
Cd36	AGATGACGTGGCAAAGAACAG	CCTTGGCTAGATAACGAACTCTG
Cd68	TGTCTGATCTTGCTAGGACCG	GAGAGTAACGGCCTTTTTGTGA
Cebpa	CAAGAACAGCAACGAGTACCG	GTCACTGGTCAACTCCAGCAC
Chrebp	CAGCTGCGGGATGAGATTGA	AAACGCTGGTGTGTGATGGGTA
Cidea	TGACATTCATGGGATTGCAGAC	GGCCAGTTGTGATGACTAAGAC
Cyp2e1	CGTTGCCTTGCTTGTCTGGA	AAGAAAGGAATTGGGAAAGGTCC
Dgat1	TCCGTCCAGGGTGGTAGTG	TGAACAAAGAATCTTGCAGACGA
F4/80	GGATGTACAGATGGGGGATG	CATAAGCTGGGCAAGTGGTA
Fasn	TCCTGGAACGAGAACACGATCT	GAGACGTGTCACTCCTGGACTTG
Gpam	CAACACCATCCCCGACATC	GTGACCTTCGATTATGCGATCA
Gpx1	AGTCCACCGTGTATGCCTTCT	GAGACGCGACATTCTCAATGA
Hmgcs1	GACAAGAAGCCTGCTGCCATA	CGGCTTCACAAACCACAGTCT
Hmgcr	ATATAGCTCGTGGAATGGCAAT	AATGTCATGAACACAAAGTAGTTGG
Khka	CCAACATTCTGTGGACTTACG	CCTTCTCAAAGTCCTTAGCAG
Khkc	GCTGACTTCAGGCAGAGG	CCTTCTCAAAGTCCTTAGCAG
Ldlr	GAGGAACTGGCGGCTGAA	GTGCTGGATGGGGAGGTCT
Lipe	CAGAAGGCACTAGGCGTGATG	GGGCTTGCGTCCACTTAGTTC
Lpin1	GCTCCCGAGAGAAAGTGGTGGA	GGCTTTCCATTCTCGCAGCTCCT
Lpin2	AGTTGACCCCATCACCGTAG	CCCAAAGCATCAGACTTGGT
Mmp9	CTGGACAGCCAGACACTAAAG	CTCGCGGCAAGTCTTCAGAG
Mttp	CTCTTGGCAGTGCTTTTTCTCT	GAGCTTGTATAGCCGCTCATT

Pnpla2	CAACGCCACTCACATCTACGG	GGACACCTCAATAATGTTGG
Pnpla3	TCACCTTCGTGTGCAGTCTC	CCTGGAGCCCGTCTCTGAT
Ppara	CCTGAACATCGAGTGTCGAATAT	GTTCTTCTTCTGAATCTTGCAGCT
Pparg	GCCCACCAACTTCGGAATC	TGCGAGTGGTCTTCCATCAC
Pparg2	TCGCTGATGCACTGCCTATG	GAGAGGTCCACAGAGCTGATT
Ppargc1a	AGCCGTGACCACTGACAACGAG	GCTGCATGGTTCTGAGTGCTAAG
Ppia	GAGCTGTTTGCAGACAAAGTTC	CCCTGGCACATGAATCCTGG
Prdm16	CCACCAGCGAGGACTTCAC	GGAGGACTCTCGTAGCTCGAA
Scd1	TTCTTGCGATACACTCTGGTGC	CGGGATTGAATGTTCTTGTCGT
Slc2a1	Quantitect primer assays (Qiagen)	
Slc2a2	TTCCAGTTCGGCTATGACATCG	CTGGTGTGACTGTAAGTGGGG
Slc2a3	ATGGGGACAACGAAGGTGAC	GTCTCAGGTGCATTGATGACTC
Slc2a4	Quantitect primer assays (Qiagen)	
Slc2a5	TTCCAATATGGGTACAACGTAGC	GCGTCAAGGTGAAGGACTCAA
Srebf1c	GAGCCATGGATTGCACATTT	CTCAGGAGAGTTGGCACCTG
Tbp	AGAACAATCCAGACTAGCAGCA	GGGAACTTCACATCACAGCTC
Tgfb1	CACTGGAGTTGTACGGCAGTG	AGAGCAGTGAGCGCTGAATC
Ucp1	AGGCTTCCAGTACCATTAGGT	CTGAGTGAGGCAAAGCTGATTT
Vegfa	CACGACAGAAGGAGAGCAGAAGT	TTCGCTGGTAGACATCCATGAA

Supplementary Table 2. Cholesterol synthesis and absorption biomarkers in the liver.

	ng/mg of liver			10^2 µg/mg of cholesterol		
	**wt**	**gt/gt**	***P***	**wt**	**gt/gt**	***P***
Cholesterol (µg/mg)	9.3 ± 0.5	8.3 ± 0.7	0.211			
Squalene	29.2 ± 1.6	27.7 ± 2.2	0.580	317.8 ± 17.2	341.4 ± 17.5	0.346
Lanosterol	5.5 ± 0.32	4.2 ± 0.3	0.004**	61.0 ± 5.0	53.2 ± 4.3	0.248
Desmosterol	5.9 ± 0.2	4.8 ± 0.3	0.005**	64.1 ± 2.0	59.2 ± 2.6	0.149
Cholestenol	1.7 ± 0.1	1.3 ± 0.1	0.016*	18.9 ± 0.8	16.0 ± 0.9	0.023*
Lathosterol	3.8 ± 0.2	2.9 ± 0.2	0.012*	41.8 ± 3.2	37.9 ± 2.3	0.327
Cholestanol	34.3 ± 2.0	26.8 ± 2.2	0.023*	353.7 ± 13.1	326.7 ± 15.0	0.187
Campesterol	23.4 ± 1.4	18.6 ± 1.7	0.042*	256.2 ± 18.7	236.6 ± 24.2	0.529
Sitosterol	4.4 ± 0.2	3.2 ± 0.2	0.0004***	48.8 ± 3.9	40.9 ± 3.3	0.132

Wild-type (wt) and *Hif-p4h-2*$^{gt/gt}$ (gt/gt) mice (n=12/group). Data are means ± SEM. **p* <0.05, ***p* <0.01, ****p* <0.001.

References

[1] Hers, H.G. (1955). Fructokinase (ketohexokinase). In: *Methods in Enzymology*, Vol 1 (S.P. Colowick and N.O. Kaplan, eds.), p.286-290, Academic Press Inc., New York, NY USA.

[2] Werman MJ, Bhathena SJ (1995) Fructose metabolizing enzymes in the rat liver and metabolic parameters: Interactions between dietary copper, type of carbohydrates, and gender. J Nutr Biochem 6:373-379.

[3] Roe, J.H. (1934). A calorimetric method for the determination of fructose in blood and urine. J. Biol. Chem. 107, 15-22.

[4] Rahtu-Korpela L, Karsikas S, Horkko S, Blanco Sequeiros R, Lammentausta E, Makela KA, Herzig KH, Walkinshaw G, Kivirikko KI, Myllyharju J et al. (2014) HIF prolyl 4-hydroxylase-2 inhibition improves glucose and lipid metabolism and protects against obesity and metabolic dysfunction. Diabetes 63:3324-3333.

[5] Laitakari A, Ollonen T, Kietzmann T, Walkinshaw G, Mennerich D, Izzi V, Haapasaari K, Myllyharju J, Serpi R, Dimova EY et al. (2019) Systemic inactivation of hypoxia-inducible factor prolyl 4-hydroxylase 2 in mice protects from alcohol-induced fatty liver disease. Redox Biol 22:101145.
